# Isolation of Neutral, Mono‐, and Dicationic B_2_P_2_ Rings by Diphosphorus Addition to a Boron−Boron Triple Bond

**DOI:** 10.1002/anie.202102218

**Published:** 2021-05-01

**Authors:** Tobias Brückner, Felipe Fantuzzi, Tom E. Stennett, Ivo Krummenacher, Rian D. Dewhurst, Bernd Engels, Holger Braunschweig

**Affiliations:** ^1^ Institute for Inorganic Chemistry Julius-Maximilians-Universität Würzburg Am Hubland 97070 Würzburg Germany; ^2^ Institute for Sustainable Chemistry & Catalysis with Boron Julius-Maximilians-Universität Würzburg Am Hubland 97070 Würzburg Germany; ^3^ Institute for Physical and Theoretical Chemistry Julius-Maximilians-Universität Würzburg Emil-Fischer-Strasse 42 97074 Würzburg Germany

**Keywords:** boron, density functional calculations, oxidation, phosphorus heterocycles, radicals

## Abstract

The NHC‐stabilised diboryne (B_2_(SIDep)_2_; SIDep=1,3‐bis(2,6‐diethylphenyl)imidazolin‐2‐ylidene) undergoes a high‐yielding P−P bond activation with tetraethyldiphosphine at room temperature to form a B_2_P_2_ heterocycle via a diphosphoryldiborene by 1,2‐diphosphination. The heterocycle can be oxidised to a radical cation and a dication, respectively, depending on the oxidant used and its counterion. Starting from the planar, neutral 1,3‐bis(alkylidene)‐1,3‐diborata‐2,4‐diphosphoniocyclobutane, each oxidation step leads to decreased B−B distances and loss of planarity by cationisation. X‐ray analyses in conjunction with DFT and CASSCF/NEVPT2 calculations reveal closed‐shell singlet, butterfly‐shaped structures for the NHC‐stabilised dicationic B_2_P_2_ rings, with their diradicaloid, planar‐ring isomers lying close in energy.

## Graphical abstract

Room temperature P−P bond addition to an NHC‐stabilised diboryne yielded a four‐membered B_2_P_2_ heterocycle, which can be further oxidised to a radical cation and a dication depending on the oxidation reagent and its counterion.

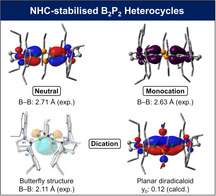

Cyclic compounds of boron and phosphorus have attracted the attention of main‐group chemists for decades.^[1]^ The early discoveries in this field focussed on oligomers of the phosphinoboranes, R_2_PBR_2_.^[2]^ In comparison to their lighter congeners, the aminoboranes, R_2_NBR_2_, the reluctance of phosphorus to adopt a planar geometry and engage in π‐bonding with boron leads to a higher propensity for intermolecular B−P association and formation of four‐membered (**A**, Scheme 1) and six‐membered heterocycles, and indeed polymers.^[3]^ A number of four‐membered 1,3‐diphospha‐2,4‐diboretanes (**B**) have also been reported,^[4]^ largely from unsuccessful attempts to prepare monomeric RP=BR species.^[5]^ These compounds contain pyramidalised phosphorus atoms that can act as ligands for transition metals.[^4e^, ^6^]

**Scheme 1:**
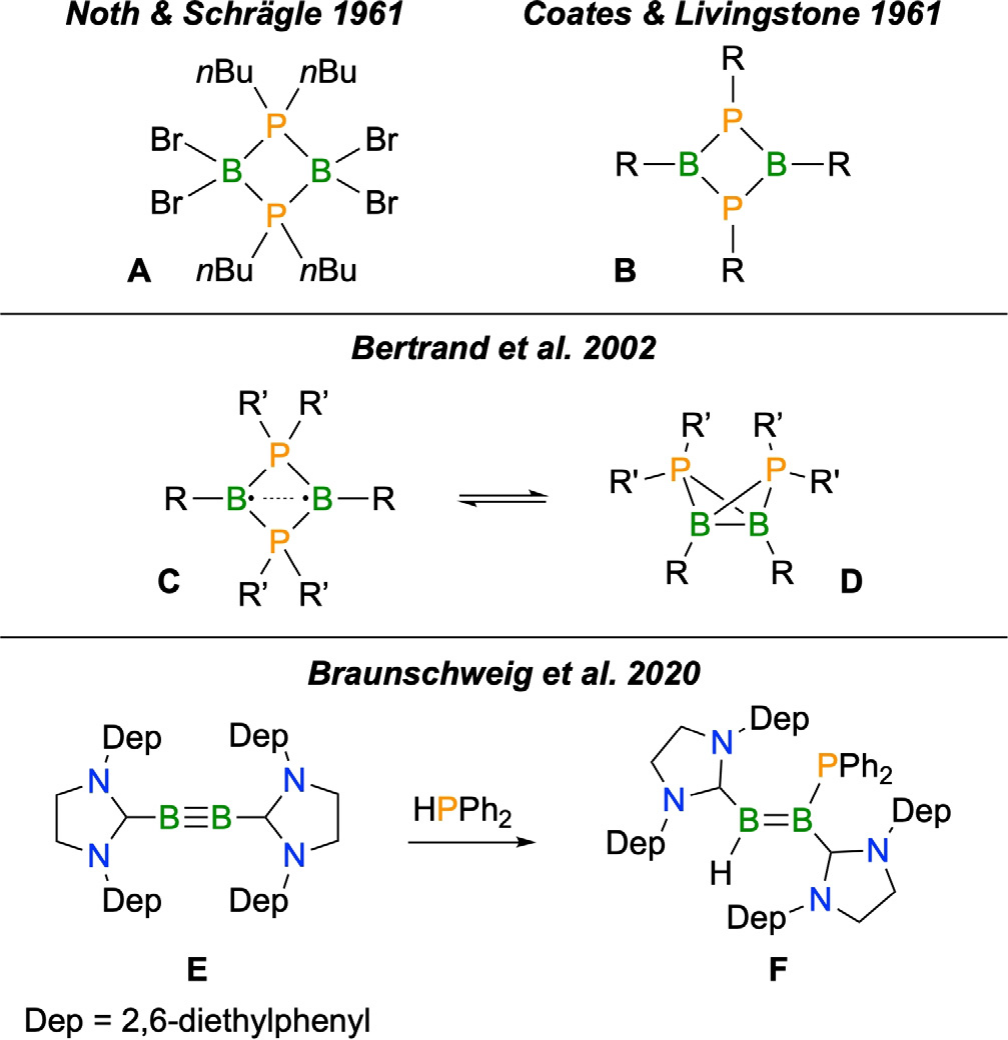
Top: relevant classes of boron–phosphorus ring compounds. Middle: synthesis of a B_2_P_2_ diradicaloid. Bottom: hydrophosphination of a diboryne.

The majority of developments in P–B ring systems in the last two decades relate to the synthesis of a 1,3‐diphospha‐2,4‐diboretanediyl diradicaloid (**C**, Scheme 1) by Bertrand and co‐workers.^[7]^ The first example of such a compound, *cyclo*‐(B*t*Bu)_2_(P*i*Pr_2_)_2_, was prepared by reacting the diborane(4) 1,2‐B_2_Cl_2_ *t*Bu_2_ with LiP*i*Pr_2_. The presumed 1,2‐diphosphinodiborane intermediate undergoes a rearrangement to cleave the B−B σ bond and generate the four‐membered ring. This compound possesses π single bond character between the two boron atoms despite the large distance between these atoms (2.57 Å), with a low‐lying B−B antibonding LUMO. Theoretical studies^[8]^ on diradicaloids **C** revealed large singlet–triplet gaps of +23.4 to +33.7 kcal mol^−1^, while the population of the LUMO from distinct two‐electron‐in‐two‐orbital approaches was found to be 0.169 to 0.19 electrons, indicating weak but distinct diradical character. This was supported by examples of diradical‐like reactivity,^[9]^ including reactions with Me_3_SnH and BrCCl_3_. Other derivatives of **C** were later prepared, with the conclusion that such compounds have two structural isomers^[10]^—the aforementioned planar diradicaloids and butterfly‐type structures containing a conventional boron−boron σ bond (**D**, Scheme 1)—which can interconvert with the appropriate selection of substituents.^[10a]^

For several years, our group has been interested in the chemistry of low‐valent boron species stabilised by strong Lewis bases.^[11]^ Of particular interest are compounds with boron−boron multiple bonds,^[12]^ diborenes and diborynes, which are able to reductively cleave a wide range of polar^[13]^ and non‐polar^[14]^ bonds. We recently used this methodology to develop a new route to covalent boron−phosphorus bonds, namely the hydrophosphination of diborenes and diborynes.^[15]^ This work included the catalyst‐free reaction of the diboryne B_2_(SIDep)_2_ (**E**, SIDep=1,3‐bis(2,6‐diethylphenyl)imidazolin‐2‐ylidene) with HPPh_2_ to yield a hydro(phosphino)diborene (**F**, Scheme 1). We recognised herein a potential opportunity to prepare B_2_P_2_ compounds, related to **C** and **D** by addition of two further valence electrons, via the activation of a phosphorus−phosphorus bond.

As a starting point, we again chose diboryne **E** due to the relatively low steric demand of the flanking carbenes compared to other derivatives. Treatment with the diphosphane P_2_Et_4_ produced a gradual colour change from red to dark green. After 6 h, a new signal was observed at 33 ppm in the ^11^B NMR spectrum in an approximate 1:1 ratio with unreacted starting material. This is indicative of diborene formation (the equivalent P‐substituted boron atom in **F** displays a resonance at 38 ppm) and suggests that the expected 1,2‐diphosphination across the B−B bond had occurred to yield compound **1** (Figure 1).

**Figure 1:**
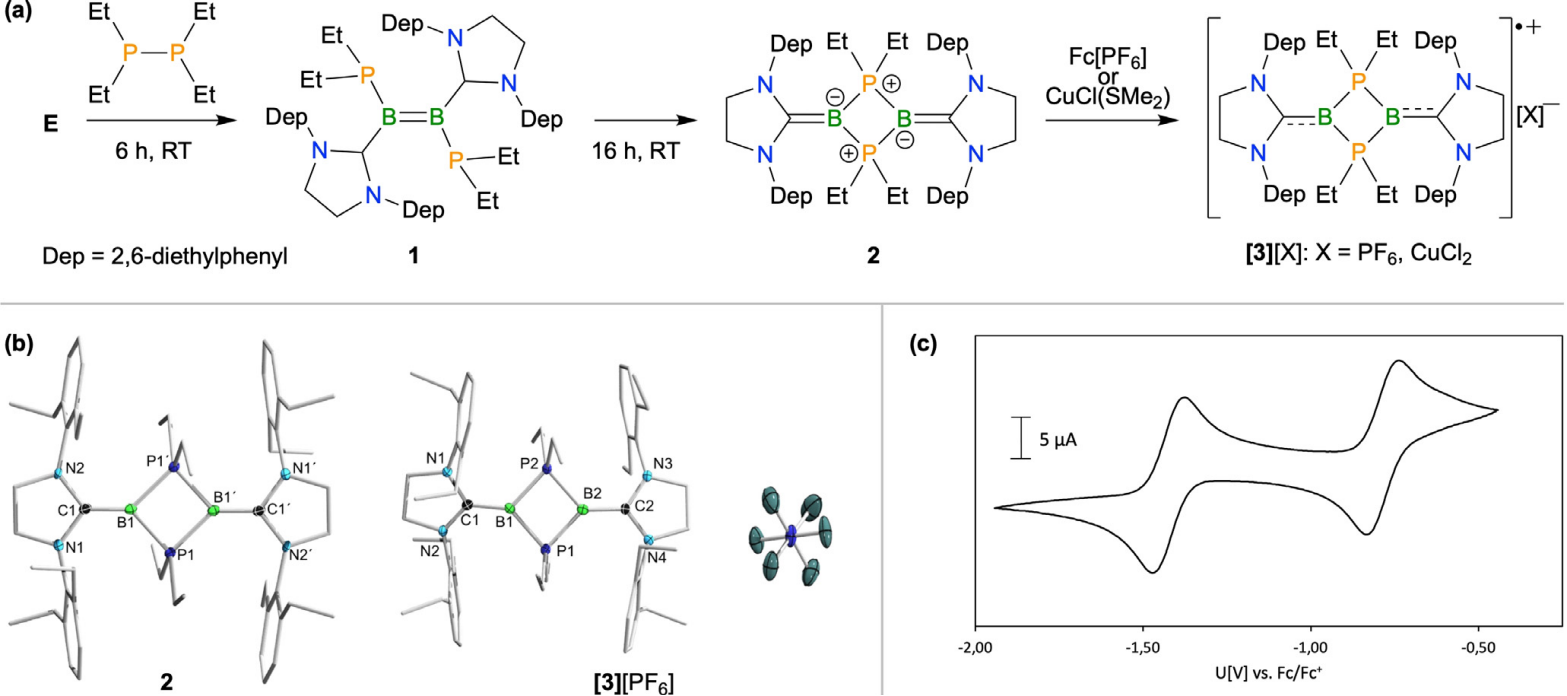
a) Synthesis of B_2_P_2_ heterocycle **2** and salts of the radical cation **[3]** ^+.^. b) Molecular structures of **2** (left) and the cationic part of **[3]**[PF_6_] (right) with atomic displacement ellipsoids at the 50 % probability level.^[22]^ Selected bond lengths [Å] and (torsion) angles [°] for **2**: B1‐B1′ 2.713(3), B1‐P1 1.913(2), B1‐P1′ 1.917(2), C1‐B1 1.468(3), N2‐C1 1.412(3), N1‐C1 1.406(3), P1‐B1‐P1′ 89.8(1), B1‐P1‐B1′ 90.2(1), C1‐B1‐B1′‐C1′ 180.0(1), N1‐N2‐N1′‐N2′ 180.0(1); for **[3]**[PF_6_]: B1‐B2 2.632(5), B1‐P1 1.905(3), B1‐P2 1.902(3), B2‐P1 1.906(3), B2‐P2 1.906(3), C1‐B1 1.528(4), B2‐C2 1.534(4), N1‐C1 1.385(4), N2‐C1 1.360(4), C2‐N3 1.375(4), C2‐N4 1.358(4), P1‐B1‐P2 92.7(2), B1‐P1‐B2 87.4(2), B1‐P2‐B2 87.3(2), P1‐B2‐P2 92.5(2), C1‐B1‐B2‐C2 48.0(1), N1‐N2‐N3‐N4 37.7(1). c) Cyclic voltammogram of **2** in THF/0.1 m [*n*Bu_4_N][PF_6_] measured with a feed rate of 250 mV s^−1^. Formal potentials: *E*_1/2_=−1.45 V, *E*_1/2_=−0.76 V (relative to Fc/Fc^+^).

A slightly broadened new signal at −44 ppm in the ^31^P NMR spectrum alongside unreacted P_2_Et_4_ (*δ*=−33 ppm) was also consistent with this assignment. The mixture was left to stand at room temperature for a further 12 h. Rather than the expected complete conversion to **1**, a further colour change to orange had occurred, accompanied by selective generation of a new set of NMR signals. While a broad signal in the ^31^P NMR at −9.6 ppm suggested a significantly different phosphorus environment, a triplet resonance at −18.9 ppm (^1^ *J*_BP_=125 Hz) in the ^11^B NMR was more revealing, suggesting bonding between boron and two equivalent phosphorus atoms. Slow evaporation of a hexane solution of the compound provided red crystals suitable for X‐ray diffraction, allowing confirmation of its identity as 1,3‐bis(alkylidene)‐1,3‐diborata‐2,4‐diphosphonio‐cyclobutane **2** (Figure 1 b). The B_2_P_2_ ring is an almost perfect square, and lies on a crystallographic inversion centre. The B−P bonds are of equal length within experimental error (1.913(2), 1.917(2) Å) and the internal angles at boron (P‐B‐P=89.8(1)°) and phosphorus (B‐P‐B=90.2(1)°) are right angles. The N‐heterocyclic carbene units are also planar with respect to the central ring, and the short B−C bond of 1.468(3) Å is in the range of a double bond rather than a dative interaction. Attempts to crystallise compound **1** eventually produced a single crystal of sufficient quality to confirm its connectivity by X‐ray diffraction (see SI), but unfortunately the data are insufficient for discussion of structural parameters.

The B_2_P_2_ ring in **2** has two electrons more than that in **D**, on account of the donor capability of the NHC units, which leads to a completely closed‐shell system. Nevertheless, their structural similarities are striking. The distance between the two boron atoms is larger in **2**, at 2.713(3) Å, indicating even less bonding interaction between these atoms than in **D**. In order to better understand the bonding in these B_2_P_2_ rings, DFT calculations at the B3LYP‐D3(BJ)/def2‐SVP level of theory^[16]^ were performed (Figure 2). As expected from the influence of the NHC substituents, the HOMO (LUMO) of **D** correlates with the HOMO−1 (HOMO) of **2**. The HOMO−1 of **2** describes a formally π‐bonding orbital derived predominantly from the p orbitals of the boron atoms, with a small degree of overlap. A small amount of delocalisation to the carbon atoms of the NHCs distinguishes this orbital from the HOMO of **D**. The HOMO of **2** represents the mutually out‐of‐phase B=C π bonds and is weakly π antibonding with respect to the two boron atoms. The population of both bonding and antibonding π(BB) orbitals results in a Mayer bond order (MBO)^[17]^ for the B−B interaction of <0.05, in contrast to **D**, which shows a substantial degree of B−B π bonding (MBO=0.274). The MBOs of 1.600 and 1.592 for the B−C bonds of **2** clearly indicate significant double bond character, while the P−B bonds (MBO=0.974–0.988) are single bonds.

**Figure 2:**
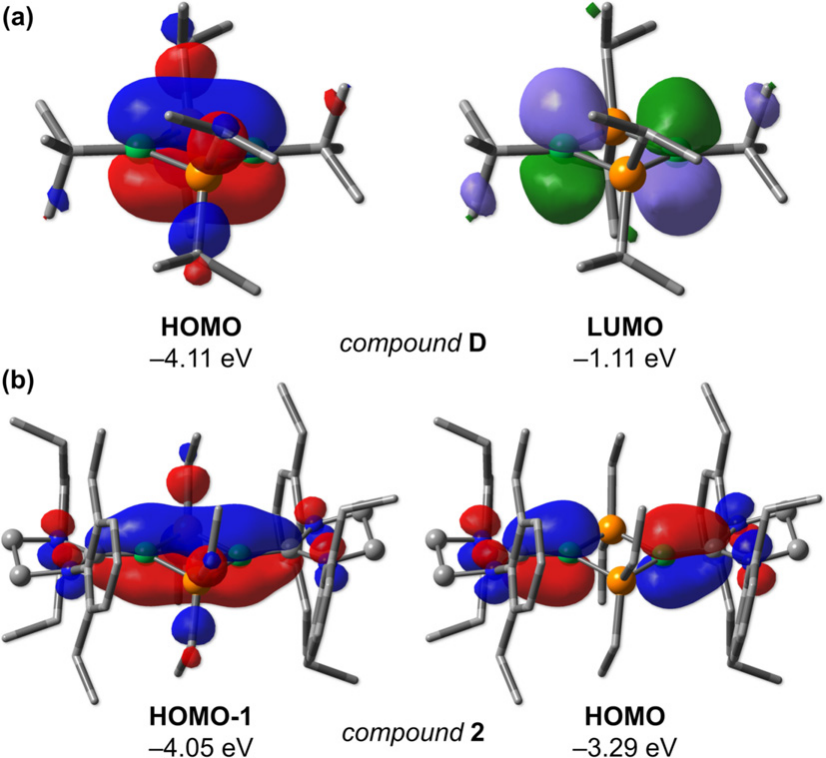
Selected frontier MOs of a) **D** and b) **2** at B3LYP‐D3(BJ)/def2‐SVP.

In light of these insights into the electronic structure of **2**, we anticipated that the compound should undergo facile oxidation. Indeed, cyclic voltammetry experiments revealed two distinct, seemingly reversible oxidation events at −0.76 and −1.45 V vs. Fc/Fc^+^ (Figure 1 c). The first oxidation is attributed to a single‐electron process leading to a radical cation, while the second oxidation would be expected to lead to a singlet diradicaloid of the form **D** or its corresponding closed‐shell singlet butterfly‐shaped counterpart. The apparent reversibility of these two redox processes is plausible as the B−B bond that is formed/broken is very weak and is accompanied by relatively minor structural changes (i.e. bending of the B_2_P_2_ ring).

We subsequently attempted to oxidise compound **2** on a preparative scale. Treatment with an equimolar amount of the weak oxidising agent [Fe(η^5^‐C_5_H_5_)_2_][PF_6_] in C_6_D_6_ solution led to a rapid colour change from orange to pink and the disappearance of all signals in the NMR spectra, suggesting the formation of a paramagnetic species (**[3]**[PF_6_], Figure 1). The EPR spectrum of **[3]**[PF_6_] (see Supporting Information) exhibits a broad resonance at *g=*2.0025 which is dominated by a 1:2:1 triplet created by a large hyperfine coupling to the two phosphorus nuclei (*a*(^31^P)=51 MHz). After several hours, red crystals formed in the solution, allowing the isolation of the radical cation **[3]**[PF_6_] and the confirmation of its structure by X‐ray diffraction (Figure 1 b). The structure of the B_2_P_2_ ring is slightly perturbed from that of the neutral precursor **2**, with a shortened B−B distance of 2.632(5) Å and slightly wider internal angles at boron (ca. 92.5°). The most significant difference to the neutral compound can be observed in the bonding of the SIDep moieties. The B−C bonds (B1−C1=1.528(4), B2−C2=1.534(4) Å) are 0.06–0.07 Å longer than those in **2**, while the NHCs now display a slightly twisted orientation towards the central ring (torsion angles between N‐C‐N and P‐B‐P planes=19.5° and 16.5°). Both of these parameters indicate a reduction in B−C π bonding consistent with the removal of an electron from the HOMO of **2**. The shortening of the B−B distance can also be rationalised by the removal of electron density from this orbital, which is weakly B−B antibonding in character. We conducted DFT calculations of the cation **[3]** ^+.^ to further investigate this system. Its frontier orbitals are very similar to those in **2**, with the singly‐occupied SOMO representing the B−C π bond and the weakly repulsive through‐space B−B interaction, while the HOMO represents the in‐phase C‐B‐B‐C π interaction. The MBO values are consistent with removal of an electron from the HOMO of **2**, with a reduction in the B−C bond order to 1.344/1.357 and a marginal increase of the B−B bond order to 0.054. It should be noted that the combination of **2** with [CuCl(SMe_2_)] provided the analogous CuCl_2_ salt of the radical cation, **[3]**[CuCl_2_]. The salt **[3]**[CuCl_2_] showed an EPR spectrum with essentially identical parameters to those of **[3]**[PF_6_] (see Supporting Information).

Despite the electrochemical evidence implying a second oxidation step, forming the putative dication of **2**, employment of an excess of [Fe(η^5^‐C_5_H_5_)_2_][PF_6_] or CuCl(SMe_2_) led to no further reaction. Use of two equivalents of even stronger oxidation reagents, Ag[Al(OC(CF_3_)_3_)_4_] or Ag[BAr^F^_4_] (BAr^F^_4_=B(3,5‐CF_3_‐C_6_H_3_)_4_) in [D_8_]THF led to an immediate colour change from orange to green. The ^11^B and ^31^P NMR spectra showed new signals at −1.4 and −25.5 ppm, respectively. The corresponding ^1^H NMR spectrum indicated clean conversion, whereby both products (**[4]**[Al(OC(CF_3_)_3_)_4_]_2_ and **[4]**[BAr^F^_4_]_2_) could be isolated quantitatively. While **[4]**[Al(OC(CF_3_)_3_)_4_]_2_ remains oily, even at −35 °C, **[4]**[BAr^F^_4_]_2_ crystallises as yellow crystals from a saturated Et_2_O solution. Single‐crystal X‐ray diffraction confirmed the formation of the dication **[4]**[BAr^F^_4_]_2_ (Figure 3 b) and revealed a butterfly‐type structure. Compared to the monocation of **[3]**[PF_6_], the B−B distance had decreased to 2.115(3) Å, with an angle between the two BBP planes of 64°. This B−B distance remains larger than those found for the butterfly‐type derivatives of **D** (Scheme 1), in which B−B distances as short as 1.8 Å have been observed.^[10a]^ In addition, while all P−B bond lengths of the central motif and the imidazole C−N bond lengths are shortened, the B−C bonds are elongated compared to those of **[3]**[PF_6_] (B1−C1=1.594(3) Å; B2−C2=1.591(3) Å).

**Figure 3:**
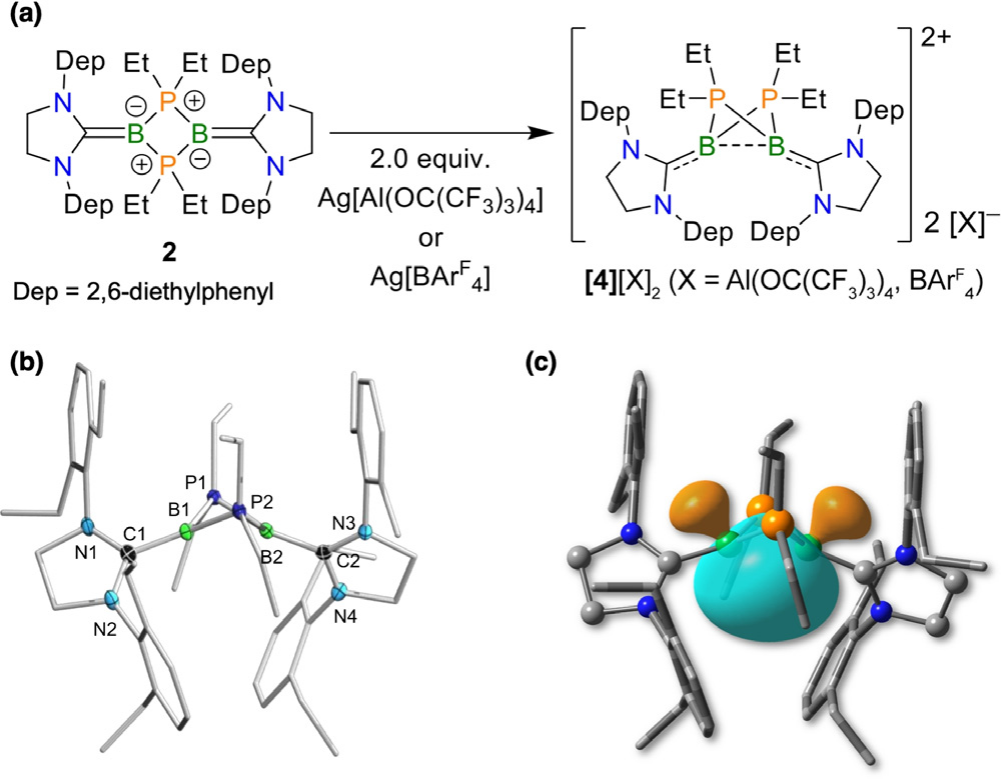
a) Formation of salts of dication **[4]** ^2+^ by double oxidation of **2**. b) Molecular structure of the dication of **[4]**[BAr^F^_4_]_2_ with atomic displacement ellipsoids at the 50 % probability level.^[22]^ Counteranions are omitted for clarity. Selected bond lengths [Å] and (torsion) angles [°]: B1‐B2 2.115(3), B1‐P1 1.884(2), B1‐P2 1.873(2), B2‐P1 1.877(2), B2‐P2 1.875(2), C1‐B1 1.594(3), B2‐C2 1.591(3), N1‐C1 1.344(2), N2‐C1 1.327(2), C2‐N3 1.347(3), C2‐N4 1.27(3), P1‐B1‐P2 96.7(2), B1‐P1‐B2 68.4(2), B1‐P2‐B2 68.7(2), P1‐B2‐P2 96.9(2), C1‐B1‐B2‐C2 31.3(1), N1‐N2‐N3‐N4 83.6(1). c) NBO of **[4]** ^2+^ depicting its bent B−B σ bond.

UV/Vis spectroscopy was performed on one example of each of the different charge states of the B_2_P_2_ systems prepared herein, namely the neutral **2** (*λ*_max_=506 nm), the cationic **[3]**[PF_6_] (*λ*_max_=538 nm) and the dicationic **[4]**[BAr^F^_4_]_2_ (*λ*_max_=639 nm). A modest redshift was observed between the neutral and monocation species (32 nm), with a more substantial redshift between the mono‐ and dications (101 nm). Assuming that these absorptions correspond to HOMO–LUMO (for **2** and **[4]**[BAr^F^_4_]_2_) or SOMO–LUMO transitions (for **[3]**[PF_6_]), these results are qualitatively in line with the initial removal of one electron from a filled orbital followed by removal of an unpaired electron, thus leaving a vacant orbital.

Natural bond orbital (NBO)^[18]^ analyses on the naked butterfly‐type dication **[4]** ^2+^ (Figure 3 c) and the corresponding system of Bertrand, **D** (R=*t*Bu, R′=*i*Pr, see Figure S12), showed that in both cases a bent^[19]^ B−B σ bond (MBO=0.472) is formed, which leads to closed‐shell singlet systems. Steric effects related to the bulky substituents of **[4]** ^2+^ prevent the boron atoms from binding at a shorter distance, affecting the system's overall stability. Consequently, the fully optimised, planar diradicaloid isomer of **[4]** ^2+^, herein labelled ^planar^ **[4]** ^2+^, is merely 3 kcal mol^−1^ higher in energy than **[4]** ^2+^ at the DFT level. Moreover, in contrast to its butterfly‐type isomer, multireference CASSCF^[20]^/NEVPT2^[21]^ calculations on ^planar^ **[4]** ^2+^ predicted a LUMO occupation of 0.41 electrons and a singlet–triplet gap of 13.3 kcal mol^−1^, which explains the two‐fold diradical character of ^planar^ **[4]** ^2+^ (y_0_=0.12) in comparison to that of **C** (R=*t*Bu, R′=*i*Pr, y_0_=0.06; Scheme 1). As the HOMO of ^planar^ **[4]** ^2+^ features the trans‐annular π‐bonding characteristic of planar derivatives of **C**,^[10a]^ barrierless thermal ring closure/opening processes that interconvert **[4]** ^2+^ and ^planar^ **[4]** ^2+^ are expected, which might allow both isomers to coexist in solution. Future work in our group will focus on the investigation of this bond‐stretch^[10a]^ isomerism between butterfly‐type and planar diradicaloid structures in novel Lewis base‐stabilised B_2_P_2_ rings.

In summary, we have developed a synthetic strategy to prepare the first NHC‐stabilised *cyclo*‐B_2_P_2_ compounds by the addition of a phosphorus−phosphorus bond to a boron−boron triple bond of a diboryne. In contrast to demonstrated reactions of other apolar E−E bonds to NHC‐stabilised diborynes,^[14]^ the 1,2‐addition product of a diphosphine, namely diphosphinodiborene **1**, rearranges to form a product with a B_2_P_2_ heterocyclic core, **2**. Compound **2** is prone to oxidation to either a radical cation or a dication, depending on the nature of the reactants and counterions. The L_2_B_2_P_2_ core of compound **2** is highly planar, with oxidation processes leading to planarity loss and decreased boron−boron distances. The dicationic species **[4]**[Al(OC(CF_3_)_3_)_4_]_2_ and **[4]**[BAr^F^_4_]_2_ have butterfly‐type structures, with their planar diradicaloid isomers lying close in energy. Our findings open new avenues for the experimental realisation of unprecedented cyclic compounds of boron and phosphorus with enhanced diradical character.

## Conflict of interest

The authors declare no conflict of interest.

## Supporting information

As a service to our authors and readers, this journal provides supporting information supplied by the authors. Such materials are peer reviewed and may be re‐organized for online delivery, but are not copy‐edited or typeset. Technical support issues arising from supporting information (other than missing files) should be addressed to the authors.

Supplementary
