# Supplementary material for: Isolation of Neutral, Mono‐, and Dicationic B2P2 Rings by Diphosphorus Addition to a Boron−Boron Triple Bond
Source: Angew Chem Int Ed Engl. 2021 May 1;60(24):13661–5. doi: 10.1002/anie.202102218 (PMC8252364; doi:10.1002/anie.202102218)
Supplement: Supplementary file 1 — Supplementary [file ANIE-60-13661-s001.pdf]

Angewandte Chemie
Eine Zeitschrift der Gesellschaft Deutscher Chemiker

Supporting Information

Isolation of Neutral, Mono-, and Dicationic B_2P_2 Rings by Diphosphorus Addition to a Boron−Boron Triple Bond

*Tobias Brückner, Felipe Fantuzzi, Tom E. Stennett, Ivo Krummenacher, Rian D. Dewhurst, Bernd Engels, and Holger Braunschweig**

anie_202102218_sm_miscellaneous_information.pdf

Contents

Synthetic procedures 2

NMR spectra 5

UV/Vis spectra 13

EPR spectroscopy 14

Cyclic voltammetry 15

Crystallographic details 16

Computational details 17

Cartesian coordinates 21

References 41

Synthetic procedures

General Considerations

All manipulations were performed either under an atmosphere of dry argon or *in vacuo* using standard Schlenk line or glovebox techniques. Deuterated solvents were dried over molecular sieves and degassed by three freeze-pump-thaw cycles prior to use. All other solvents were distilled and degassed from appropriate drying agents and stored under argon over activated 4 Å molecular sieves. NMR spectra were acquired on a Bruker Avance 500 NMR spectrometer (^{1}H: 500.1 MHz, ^{11}B: 160.5 MHz, ^{13}C{^{1}H}: 125.8 MHz, ^{31}P: 202 MHz) at 298 K unless otherwise stated. Chemical shifts (δ) are given in ppm and internally referenced to the carbon nuclei (^{13}C{^{1}H}) or residual protons (^{1}H) of the solvent. ^{11}B and ^{31}P NMR spectra were referenced to external standards ($BF_3 \cdot OEt_2$; 85% aq. H_3PO_4). High-resolution mass spectrometry data was obtained from a Thermo Scientific Exactive Plus spectrometer in ASAP or LIFDI mode. Microanalyses (C, H, N, S) were performed on an Elementar vario MICRO cube elemental analyzer. EPR measurements at X-band (9.85 GHz) were carried out at room temperature using a Bruker ELEXSYS E580 CW EPR spectrometer. CW EPR spectra were measured using 1 mW microwave power and 0.5 G field modulation at 100 kHz, with a conversion time of 20 ms. The spectral simulations were performed using MATLAB 8.6 (R2019a) and the EasySpin 5.2.25 toolbox.[1] UV/Vis absorption spectra were measured on a JASCO V-660 UV/Vis spectrometer or on a METTLER TOLEDO UV-vis-Excellence UV5 spectrophotometer. Cyclic voltammetry experiments were performed using a Gamry Instruments Reference 600 potentiostat. A standard three-electrode cell configuration was employed using a platinum disk working electrode, a platinum wire counter electrode, and a silver wire, separated by a *Vycor* tip, serving as the reference electrode. Formal redox potentials are referenced to the ferrocene/ferrocenium ($[Cp_2Fe]^{+/0}$) redox couple by using decamethylferrocene ($[Cp^{*}_2Fe]$; $E_{1/2} = -0.427$ V in THF) as an internal standard. Tetra-*n*-butylammonium hexafluorophosphate ($[n\text{Bu}_4\text{N}][\text{PF}_6]$) was employed as the supporting electrolyte. Compensation for resistive losses (*iR* drop) was employed for all measurements. B_2(SIDep)$_2$ (**E**)[2] were synthesized following literature procedures. Ag[BAr$^{F}_4$] (Ar^{F} = 3,5-$C_6H_3(CF_3)_2$) was prepared analogously to Ag[B(C_6F_5)$_4$][3] from $AgNO_3$ and Na[BAr$^{F}_4$].[4] Ag[Al(O(C(CF_3)$_3$)$_4$] was a generous gift from the group of Prof. Ingo Krossing.

<u>Synthesis of B_2(SIDep)$_2$(PEt$_2$)$_2$ (**2**):</u>

<u>NMR scale:</u> B_2(SIDep)$_2$ (20 mg; 29 µmol) were dissolved in C_6H_6 (0.3 mL) and treated with P_2Et_4 (0.55 mL, 0.05 M in hexane, 1.1 equiv.). Within 6 h at room temperature the solution turned from red to green and new ^{11}B NMR (33 ppm) and ^{31}P NMR (–44 ppm) signals were observed. After an additional 12 h at room temperature the solution turned orange and new NMR signals were observed at –19 (^{11}B) and –10 (^{31}P). All volutiles were removed under reduced pressure and the orange residue was washed

with hexane (3 x 0.5 mL). After drying in vacuo the product could be isolated in 62% yield. Crystals suitable for X-ray diffraction could be obtained by slow evaporation of a saturated hexane solution.

Larger scale synthesis: $B_2(SIDep)_2$ (1.50 g; 2.18 mmol), a solution of P_2Et_4 (4.5 mL, c = 0.5 mol/L, 1.1 equiv), benzene (50 mL), stirred at room temperature for 18 h, washed with hexane (1 x 10 mL) at room temperature, yield: 78%.

^{1}H NMR (500 MHz, C_6D_6): δ = 7.09 – 7.06 (m, 4H, CH_{Ar}), 7.02 – 7.00 (m, 8H, CH_{Ar}), 3.26 (s, 8H, NCH_2), 2.91 (q, 16H, $^3J_{H,H}$ = 7.5 Hz, CH_{2Et}), 1.21 (t, 24H, $^3J_{H,H}$ = 7.5 Hz, CH_{3Et}), 1.16 – 1.13 (m, 12H, CH_{3PEt}), –0.08 – –0.15 (m, 8H, CH_{2PEt}) ppm.

^{13}C{^{1}H} NMR (125.8 MHz, C_6D_6): δ = 160.1 (B=*C*, detected via HMBC), 143.8 (C_q), 143.1 (C_q), 128.6 (*C*H_{Ar}), 126.9 (*C*H_{Ar}), 126.4 (*C*H_{Ar}), 52.5 (N*C*H_2), 24.9 (*C*H_{2Et}), 21.6 (t, $^1J_{P,C}$ = 20.6 Hz, *C*H_{2PEt}), 14.5 (*C*H_{3Et}), 10.7 (*C*H_{3PEt}) ppm.

^{11}B NMR (160.5 MHz, C_6D_6): δ = 18.9 (t, $^1J_{P,B}$ = 118 Hz) ppm.

^{31}P NMR (202.5 MHz, C_6D_6): δ = 9.6 ppm.

HRMS (LIFDI): calc. ($C_{54}H_{80}B_2N_4P_2$ + H) m/z = 869.6117; found: m/z = 869.6116.

UV/Vis (benzene): λ_{max} = 507 nm.

Synthesis of $[B_2(SIDep)_2(PEt_2)_2][PF_6]$ (**[3]**$[PF_6]$):

$B_2(SIDep)_2$ (10 mg, 11.5 µmol) and $FcPF_6$ (3.8 mg, 11.5 µmol) were dissolved in C_6H_6 (0.6 mL) and placed on a tilting laboratory shaker overnight. During this time the solution changed from orange to pink, the ^{11}B and ^{31}P NMR signals disappeared and red crystals formed. These crystals were washed three times with C_6H_6 (0.5 mL) and dried in vacuo. **[3]**$[PF_6]$ was isolated in 91% yield as a red solid. Crystals suitable for X-ray diffraction were obtained from the reaction solution.

Elemental Analysis: ($C_{54}H_{80}B_2N_4P_3F_6(\cdot CH_2Cl_2)$): calc. C = 63.98 (60.13)%, H = 7.95 (7.52)%, N = 5.53 (5.10)%; found: C = 60.56%, H = 7.62%, N = 5.14%.

UV/Vis (CH_2Cl_2): λ_{max} = 538 nm.

Synthesis of $[B_2(SIDep)_2(PEt_2)_2][X]_2$ (**[4]**$[Al(OC(CF_3)_3)_4]_2$ and **[4]**$[BAr^F_4]_2$)

$B_2(SIDep)_2$ (30 mg, 43.5 µmol) and 2.0 equiv of the corresponding Ag(I) compound were mixed in Et_2O (0.5 mL), whereupon the solution changed from orange to green within seconds. NMR spectroscopic tracking showed complete consumption of all starting materials and quantitative conversion to the dications. Removal of the insoluble components by filtration and evaporation of the solvent gave the

products in 99% yield. Crystals of **[4]**[BAr$^F{}_4$]$_2$ suitable for X-ray diffraction could be obtained by slow evaporation of a saturated Et_2O solution.

NMR spectra for **[4]**[BAr$^F{}_4$]$_2$:

^{1}H NMR (400 MHz, d$_8$-THF): δ = 7.56 – 7.53 (m, 4H, C$H_{Ar,p\text{-}Dep}$), 7.39 – 7.38 (m, 8H, C$H_{Ar,m\text{-}Dep}$), 4.27 (s, 8H, NCH_2), 2.70 – 2.51 (m, 16H, CH_{2Dep}), 1.31 (t, $^3J_{H,H}$ = 7.41 Hz, 24H, CH_{3Dep}), 0.65 – 0.56 (m, 12H, PCH$_{3Et}$), 0.42 – 0.37 (m, 8H, PCH_{2Et}) ppm.

^{13}C{^{1}H} NMR (125.8 MHz, d$_8$-THF): δ = 177.3 (N*C*BN), 142.1 (*C*$_q$), 135.1 (*C*$_q$), 132.2 (*C*H$_{Ar}$), 128.7 (*C*H$_{Ar}$), 122.8 (q, $^1J_{C,F}$ = 293 Hz, CF$_3$), 54.5 (N*C*H$_2$), 20.3 (t, $^1J_{P,C}$ = 23 Hz, P*C*H$_2$), 14.2 (*C*H$_{3Dep}$), 10.2 (t, $^1J_{P,C}$ = 1.9 Hz, P*C*H$_3$) ppm. *Note*: The signals for the *C*H$_{2Dep}$ nuclei are obscured by the solvent signal.

^{11}B NMR (160.5 MHz, d$_8$-THF): δ = 1.4 ppm.

^{31}P NMR (202.5 MHz, d$_8$-THF): δ = 25.5 ppm.

^{19}F NMR (470.6 MHz, d$_8$-THF): δ = 75.8 ppm.

HRMS (LIFDI): calc. ([$C_{54}H_{80}B_2N_4P_2$][$C_{32}H_{12}BF_{24}$]) m/z = 1731.6688; found.: m/z = 1731.6697.

UV/Vis (THF): λ_{max} = 639 nm.

NMR spectra

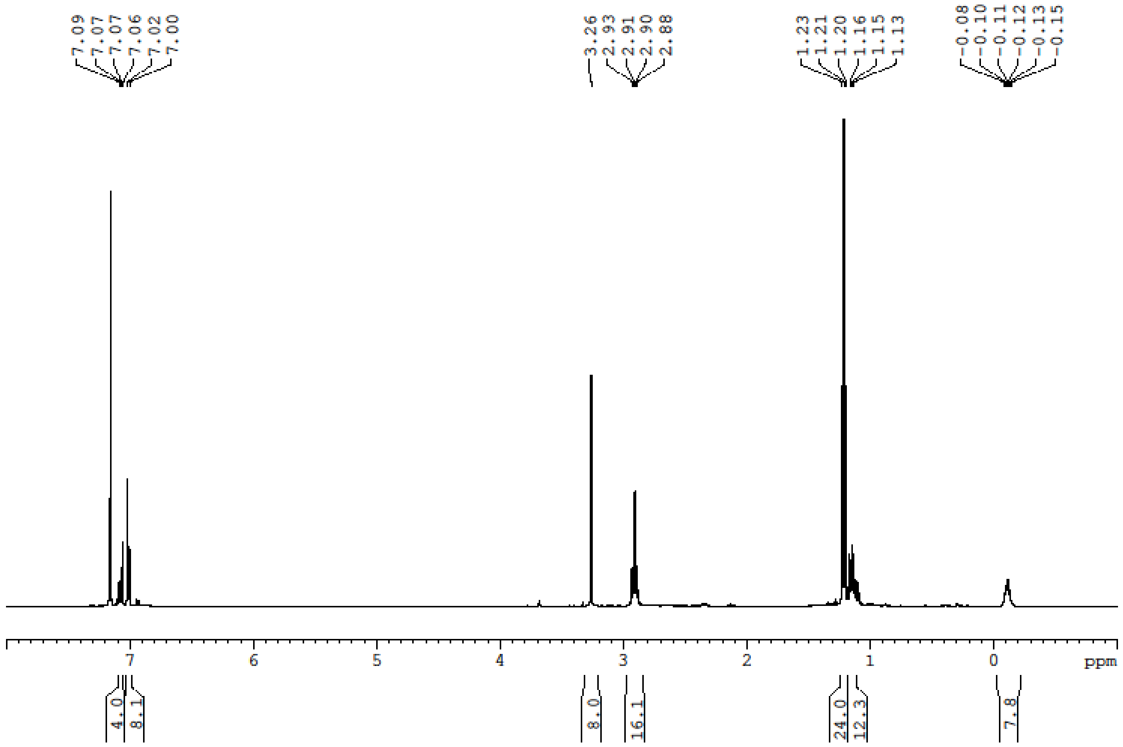

Figure S1: ^{1}H NMR spectrum of **2**.

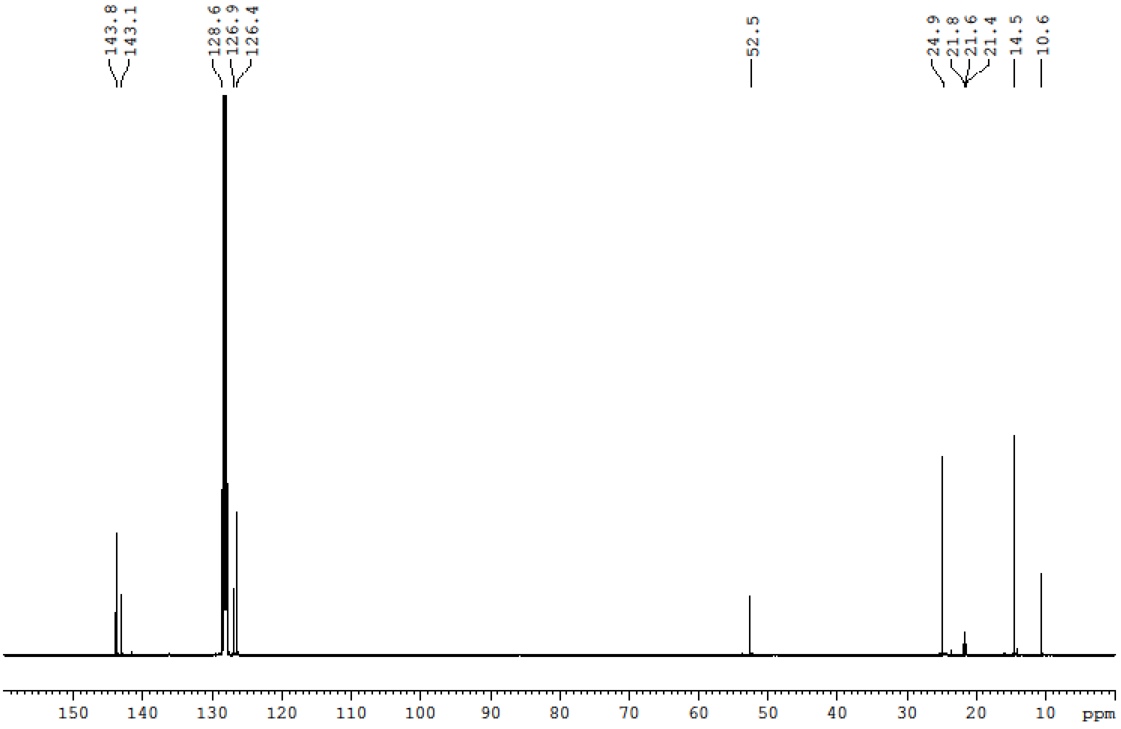

Figure S2: $^{13}C\{^{1}H\}$ NMR spectrum of **2**.

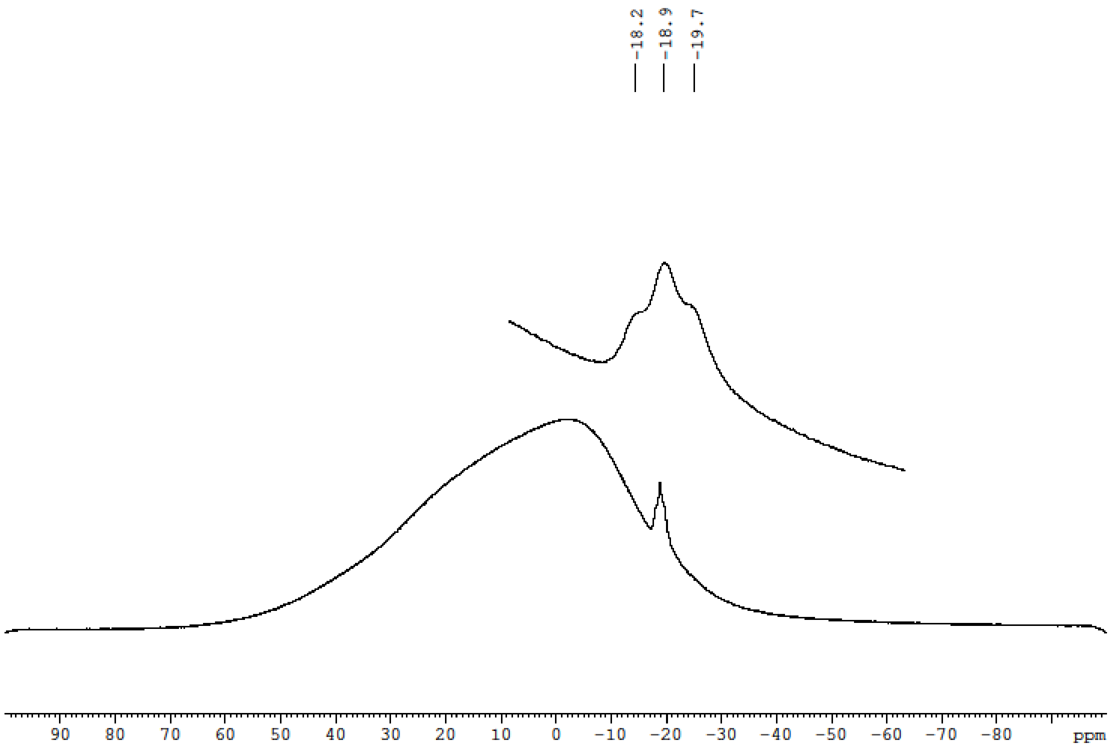

Figure S3: ^{11}B NMR spectrum of **2**.

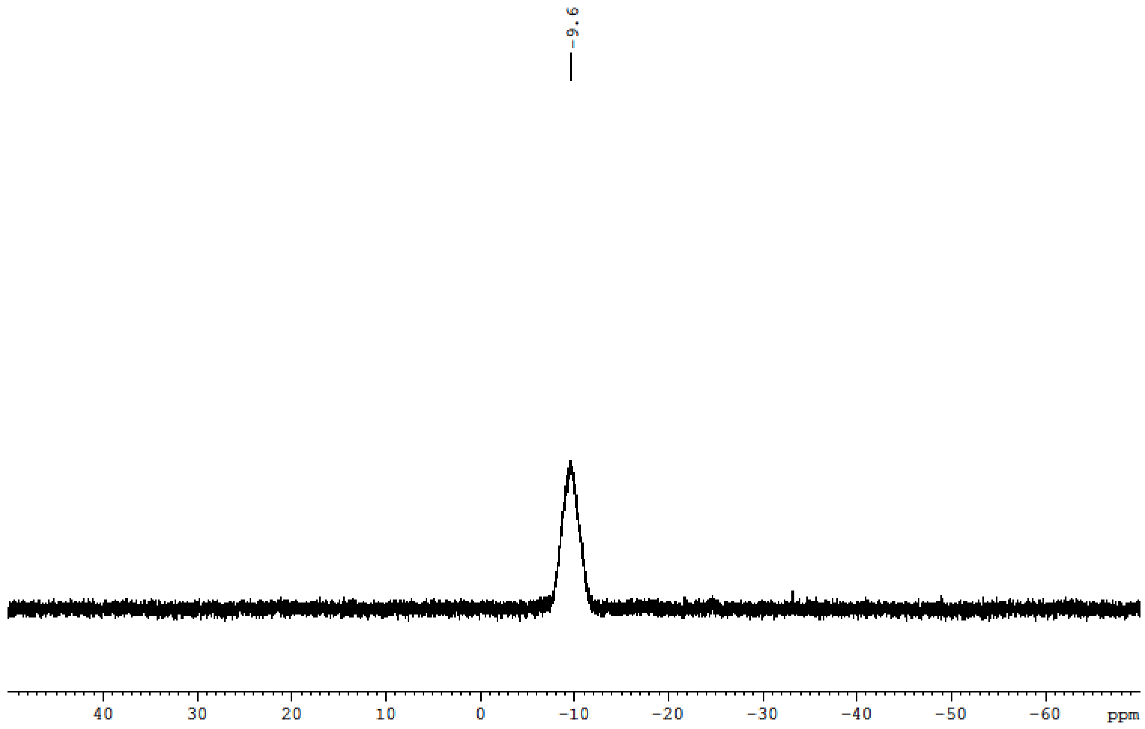

Figure S4: ^{31}P NMR spectrum of **2**.

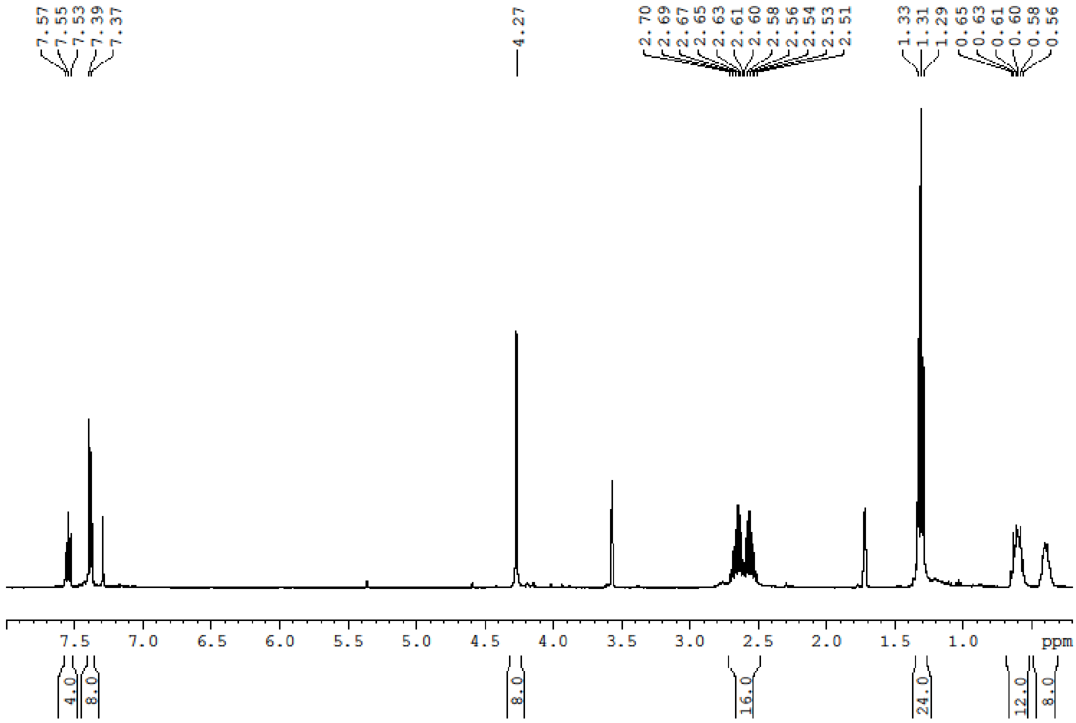

Figure S5: ^{1}H NMR spectrum of **[4]**[BAr$^F{}_4$]$_2$.

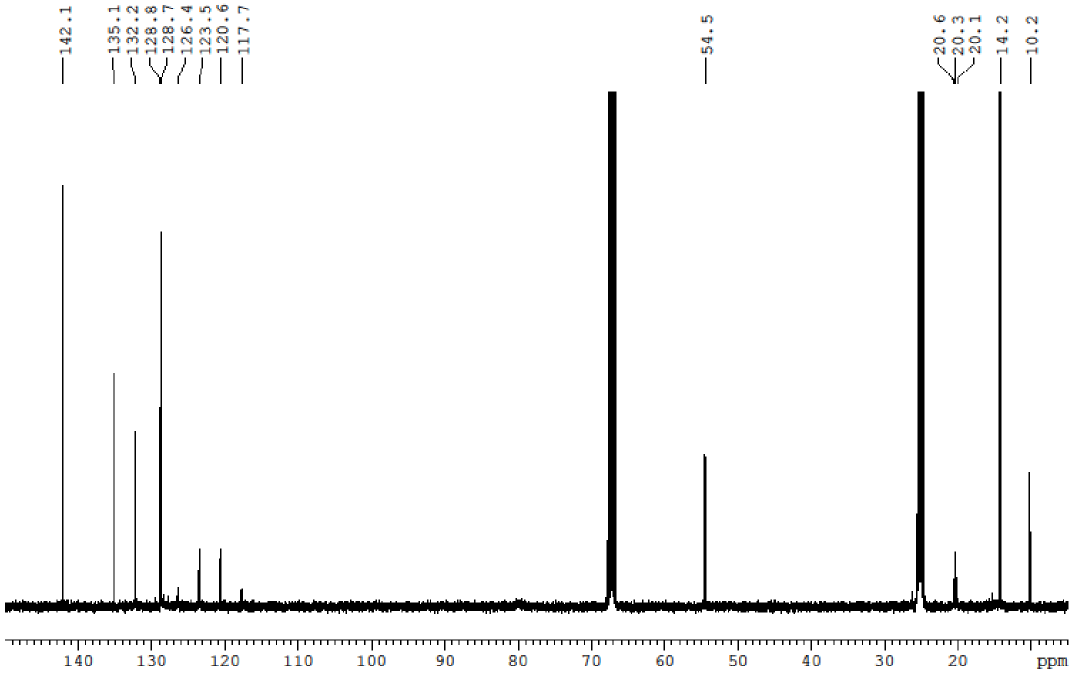

Figure S6: ^{13}C{^{1}H} NMR spectrum of **[4]**[BAr$^F{}_4$]$_2$.

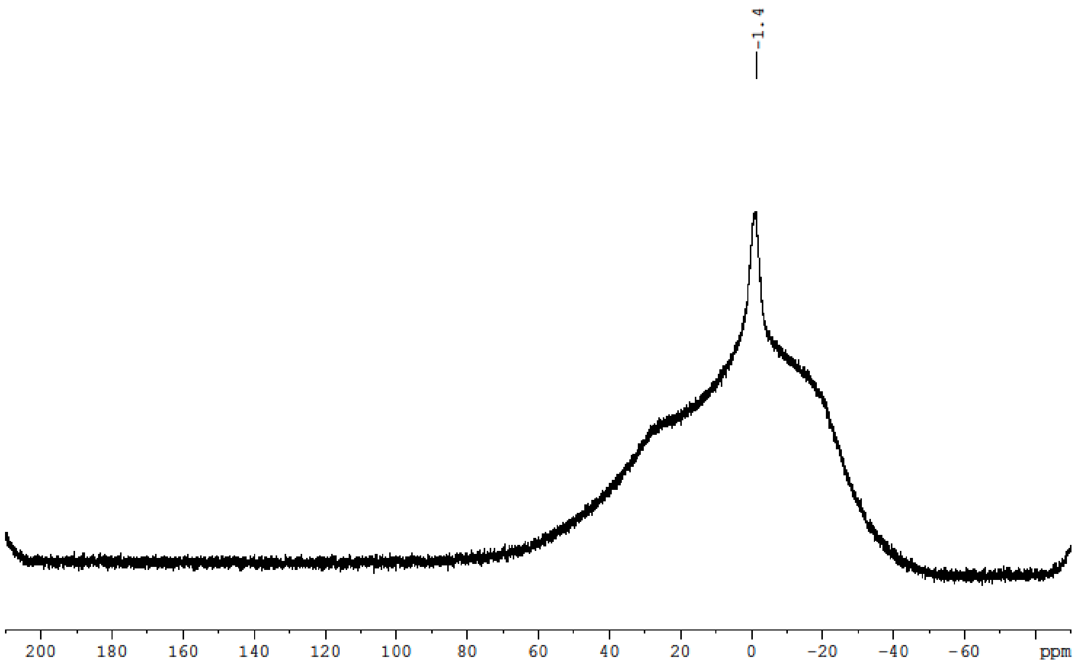

Figure S7: ^{11}B NMR spectrum of **[4]**[$BAr^F{}_4$]$_2$.

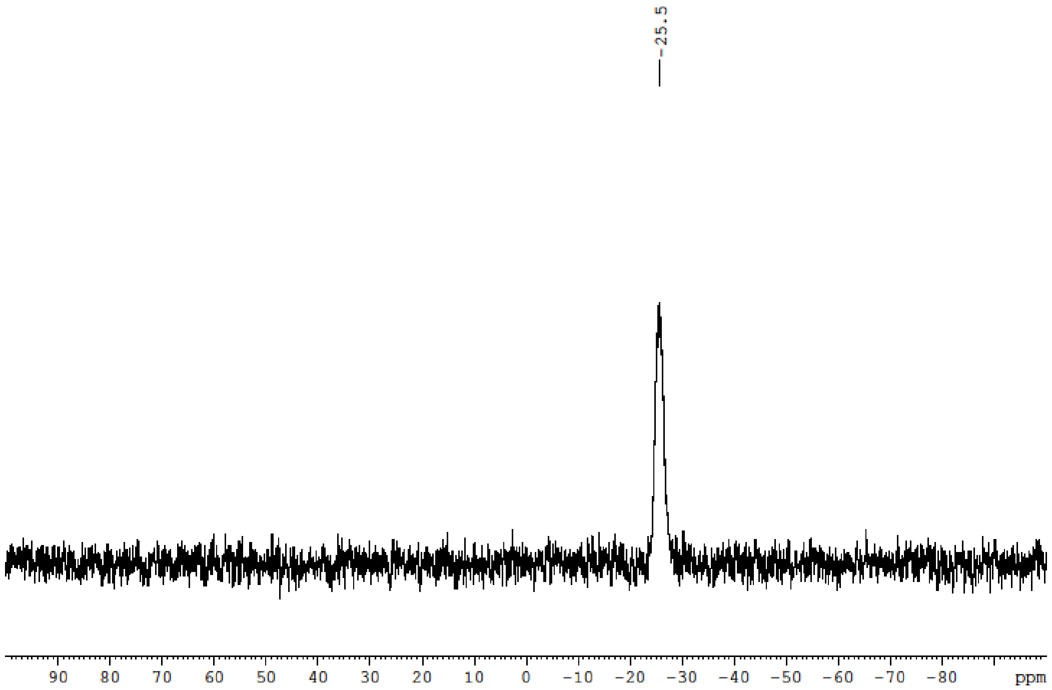

Figure S8: ^{31}P NMR spectrum of **[4]**[BAr$^{F}_{4}$]$_{2}$.

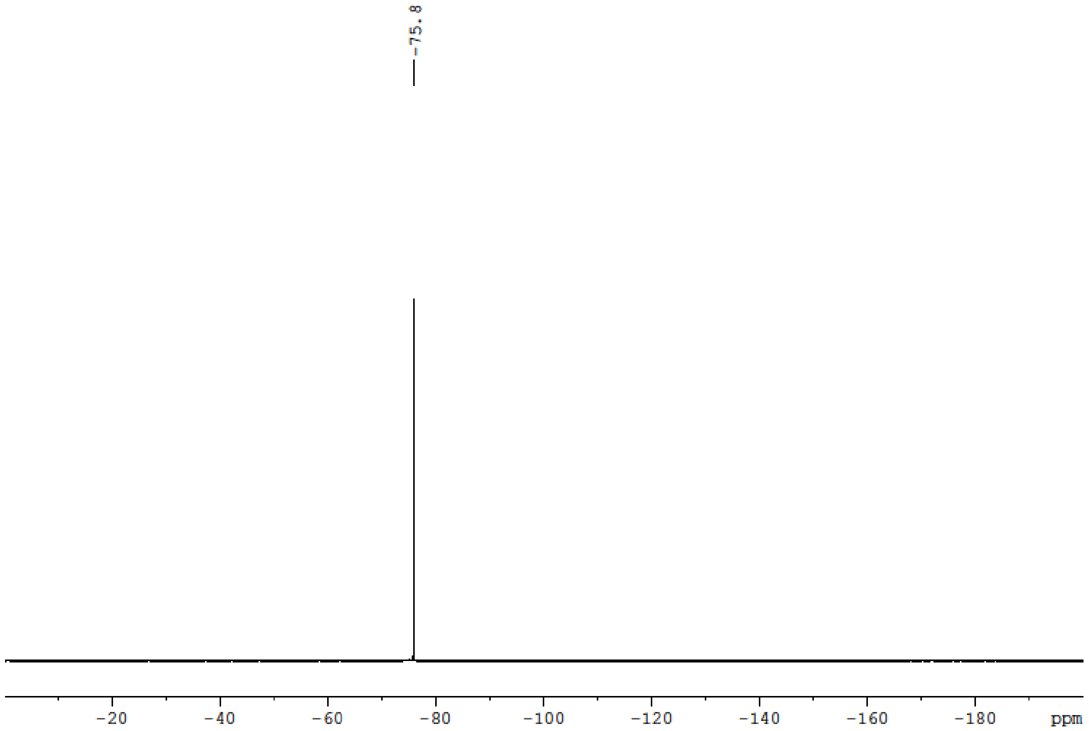

Figure S9: ^{19}F NMR spectrum of **[4]**[BAr$^{F}{}_{4}$]$_2$.

UV/Vis spectra

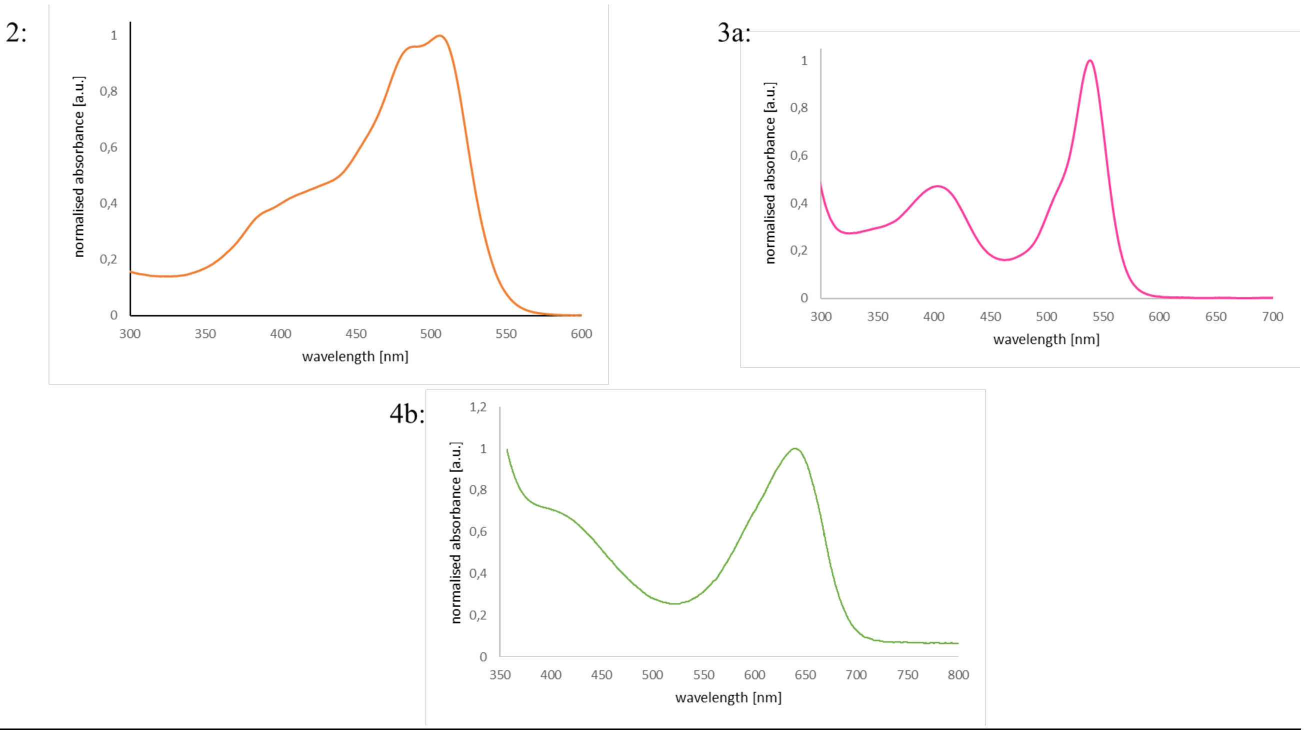

Figure S10. UV/Vis spectra of **2** (λ_{max} = 506 nm; benzene), **[3]**[PF_6] (λ_{max} = 538 nm; dichloromethane) and **[4]**[$BAr^F{}_4$]$_2$ (λ_{max} = 639 nm; tetrahydrofuran).

EPR spectroscopy

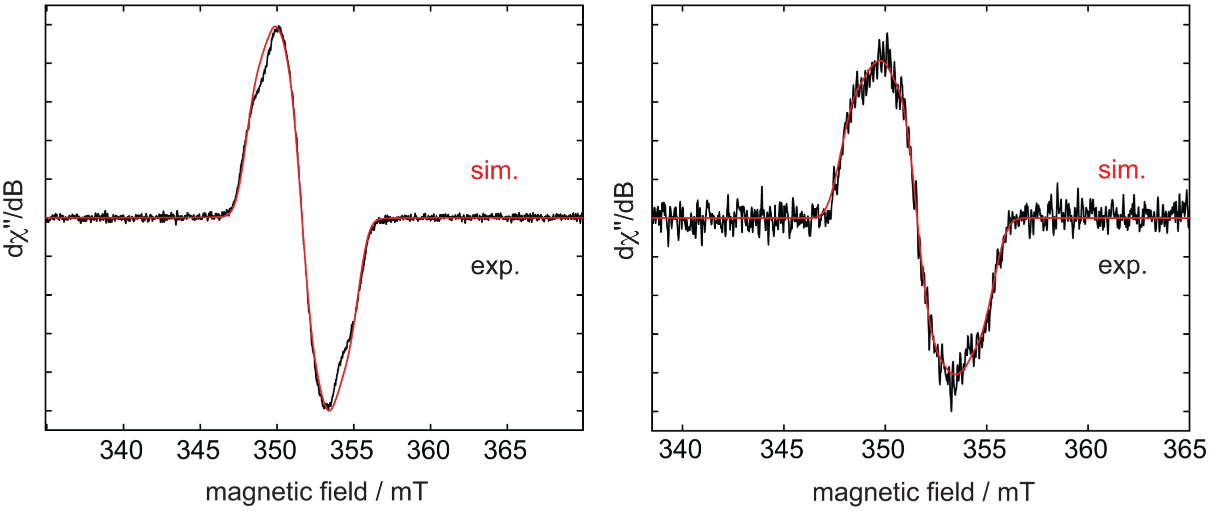

Figure S11. Right: experimental (black) and simulated (red) continuous-wave X-band EPR spectra of **[3]**[PF_6] in benzene solution at room temperature. Best-fit simulation parameters: g_{iso} = 2.0025, *a*(B) = 14.9 MHz (5.3 G), *a*(^{31}P) = 49.0 MHz (17.3 G), *a*(^{14}N) = 7.4 MHz (2.6 G). Left: experimental (black) and simulated (red) continuous-wave X-band EPR spectra of **[3]**[$CuCl_2$] in dichloromethane solution at room temperature. Best-fit simulation parameters: g_{iso} = 2.0025, *a*(B) = 15.0 MHz (5.4 G), *a*(^{31}P) = 51.0 MHz (18.2 G), *a*(^{14}N) = 7.0 MHz (2.5 G).

Cyclic voltammetry

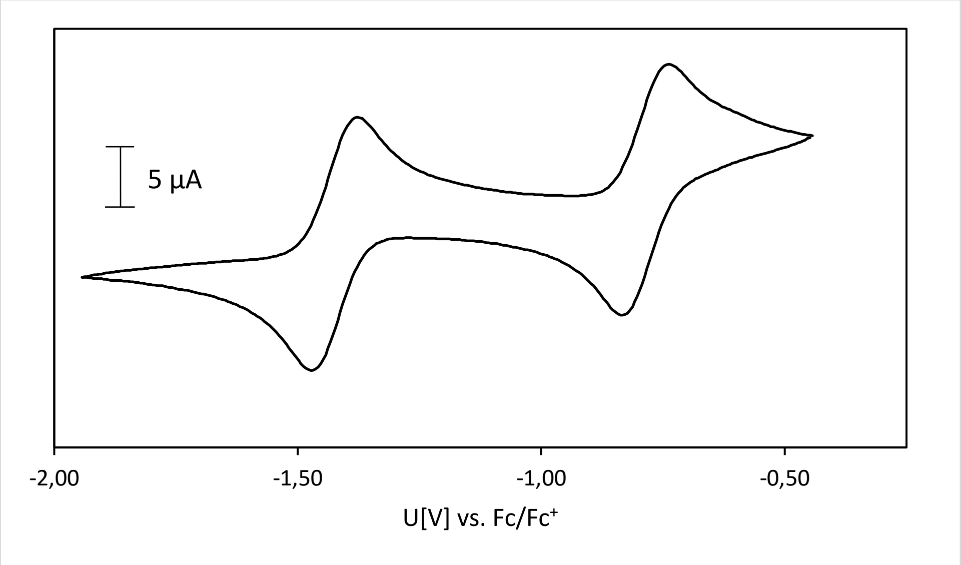

Figure S12. Cyclic voltammogram of **2** in THF/0.1 M [*n*Bu_4N][PF_6] measured with a feed rate of 250 mV/s. Formal potentials: $E_{1/2} = -1.45$ V, $E_{1/2} = -0.76$ V (relative to Fc/Fc^+).

Crystallographic details

The crystal data of **2, [3]**[PF_6], **[3]**[$CuCl_2$] and **[4]**[$BAr^F{}_4$]$_2$ were collected on a Bruker D8 Quest diffractometer with a CMOS area detector and multi-layer mirror monochromated MoK_α radiation. The structures were solved using the intrinsic phasing method,[5] refined with the SHELXL program[6] and expanded using Fourier techniques. All non-hydrogen atoms were refined anisotropically. Hydrogen atoms were included in structure factor calculations. Crystallographic data have been deposited with the Cambridge Crystallographic Data Center (CCDC numbers: 2062135-2062138). These data can be obtained free of charge from The Cambridge Crystallographic Data Centre *via* www.ccdc.cam.ac.uk/data_request/cif

Crystal data for **2**: $C_{54}H_{80}B_2N_4P_2$, $M_r = 868.78$, purple block, $0.271\times0.182\times0.143$ mm^3, monoclinic space group $C2/c$, $a = 23.243(2)$ Å, $b = 10.0064(9)$ Å, $c = 21.269(2)$ Å, $\beta = 90.798(4)°$, $V = 4946.3(9)$ Å^3, $Z = 4$, $\rho_{calcd} = 1.167$ g·cm^{-3}, $\mu = 0.128$ mm^{-1}, $F(000) = 1888$, $T = 100(2)$ K, $R_1 = 0.0835$, $wR^2 = 0.1270$, 4872 independent reflections [$2\theta \leq 52.042°$] and 306 parameters. (CCDC number: 2062135). The displacement parameters of atoms of the disordered ethyl group were restrained to the same value with similarity restraint SIMU and RIGU.

Crystal data for **[3]**[PF_6]: $C_{63}H_{89}B_2F_6N_4P_3$, $M_r = 1130.91$, pink plate, $0.204\times0.198\times0.099$ mm^3, monoclinic space group $P2_1/n$, $a = 15.2248(15)$ Å, $b = 23.035(2)$ Å, $c = 17.9777(18)$ Å, $\beta = 104.573(4)°$, $V = 6101.9(11)$ Å^3, $Z = 4$, $\rho_{calcd} = 1.231$ g·cm^{-3}, $\mu = 0.158$ mm^{-1}, $F(000) = 2416$, $T = 100(2)$ K, $R_1 = 0.1122$, $wR^2 = 0.1464$, 11616 independent reflections [$2\theta \leq 51.432°$] and 715 parameters. (CCDC number: 2062136).

Crystal data for **[3]**[$CuCl_2$]: $C_{54}H_{80}B_2Cl_2CuN_4P_2$, $M_r = 1003.22$, red plate, $0.148\times0.134\times0.076$ mm^3, orthorhombic space group $Pna2_1$, $a = 18.5991(3)$ Å, $b = 11.6574(2)$ Å, $c = 24.6400(4)$ Å, $V = 5342.38(15)$ Å^3, $Z = 4$, $\rho_{calcd} = 1.247$ g·cm^{-3}, $\mu = 0.608$ mm^{-1}, $F(000) = 2140$, $T = 104(2)$ K, $R_1 = 0.0539$, $wR^2 = 0.0881$, 10514 independent reflections [$2\theta \leq 52.044°$] and 598 parameters. (CCDC number: 2062137).

Crystal data for **[4]**[$BAr^F{}_4$]$_2$: $C_{122}H_{114}B_4F_{48}N_4OP_2$, $M_r = 2669.35$, yellow block, $0.199\times0.178\times0.167$ mm^3, monoclinic space group $C2/c$, $a = 38.287(10)$ Å, $b = 15.854(4)$ Å, $c = 40.195(13)$ Å, $\beta = 90.488(12)°$, $V = 24397(12)$ Å^3, $Z = 8$, $\rho_{calcd} = 1.453$ g·cm^{-3}, $\mu = 0.161$ mm^{-1}, $F(000) = 10912$, $T = 100(2)$ K, $R_1 = 0.0575$, $wR^2 = 0.1131$, 24022 independent reflections [$2\theta \leq 52.044°$] and 1769 parameters. (CCDC number: 2062138). The displacement parameters of atoms of the disordered Ethyl and CF_3 groups were restrained to the same value with similarity restraint SIMU and RGU. The Uii displacement parameters of atoms of the disordered CF_3 groups were restrained with the ISOR keyword to approximate isotropic behavior.

Computational details

Geometry optimizations and Hessian calculations were performed in the gas phase for **2**, **[3]**$^{+\bullet}$, **[4]**$^{2+}$, and ^{planar}**[4]**$^{2+}$, as well as for Bertrand's compounds **C** and **D** (R = *t*Bu, R' = *i*Pr), at the B3LYP[7]-D3[8](BJ)[9]/def2-SVP[10] level of theory considering restricted and unrestricted (broken-symmetry) wavefunctions. All optimized structures were characterized as minimum energy geometries as only positive eigenvalues were obtained in the vibrational frequency calculations. Stability tests[11] on the restricted and unrestricted wavefunctions were also performed, and contributed to the open-shell singlet attribution for ^{planar}**[4]**$^{2+}$. Spin density plots were obtained within the Mulliken population analysis (MPA).[12] Atomic charges were evaluated using the Hirshfeld,[13] Mulliken,[12] Löwdin,[14] atomic dipole moment corrected Hirshfeld population method (ADCH)[15] and charge model 5 (CM5)[16] approaches. Mayer bond orders (MBOs)[17] were also calculated for selected systems. Additional high-level, multireference calculations at the CASSCF[18] and NEVPT2[19] levels of theory, the latter within the strongly-contracted approach, were performed for ^{planar}**[4]**$^{2+}$ and **C** considering singlet and triplet multiplicities, to evaluate and compare the diradical character and the singlet-triplet gap of these systems. With the def2-SVP basis set, active spaces of two electrons and two orbitals, CASSCF(2,2); four electrons and four orbitals, CASSCF(4,4); and six electrons and six orbitals, CASSCF(6,6), were considered. The results were fully consistent regardless of the active space size. We, therefore, selected the CASSCF(2,2) active space and performed calculations with the triple-zeta quality def2-TZVP basis set for estimating the diradical character and singlet-triplet gaps of ^{planar}**[4]**$^{2+}$ and **C**. To speed up the multireference calculations, the resolution-of-the-identity (RI)[20] integral approximation was used for the construction of the Fock matrix and the integral generation and transformation steps involved in the CASSCF procedure. The diradical character (y_0) of **[3]**$^{+\bullet}$ was obtained using the Yamaguchi[21] formula:

$$y_i = 1 - \frac{2T_i}{1 + T_i^{\,2}} \qquad (S1)$$

where T_i is calculated from the occupation numbers (ON) of the $HOMO - i$ and $LUMO + i$ natural orbitals of a CASSCF calculation from the following expression:

$$T_i = \frac{ON_{HOMO-i} - ON_{LUMO+i}}{2} \qquad (S2)$$

For y_0, eq. S1 is reduced to:

$$y_0 = 1 - \frac{2T_0}{1 + T_0^{\,2}} \qquad (S3)$$

where T_0 is given by:

$$T_0 = \frac{ON_{HOMO} - ON_{LUMO}}{2} \qquad (S4)$$

Finally, natural bond order (NBO)[22] calculations and the bond-bending analysis were performed for **[4]**$^{2+}$, at the B3LYP/def2-SVP level, to describe its bonding situation. The NBO calculations were performed using the NBO 7.0 program.[23] Multireference calculations were performed in Orca 4.1.1.[24] MBOs and charge analyses were obtained using Multiwfn 3.7.[25] All other calculations were performed using Gaussian 16, Revision B.01.[26]

Table S1. Atomic charges on the B_2P_2 ring of **2** from distinct population analysis.

	B	P
**Hirshfeld**	–0.17	+0.22
**Mulliken**	–0.49	+0.18
**Löwdin**	–0.72	+0.83
**ADCH**	–0.14	+0.15
**CM5**	–0.32	+0.18

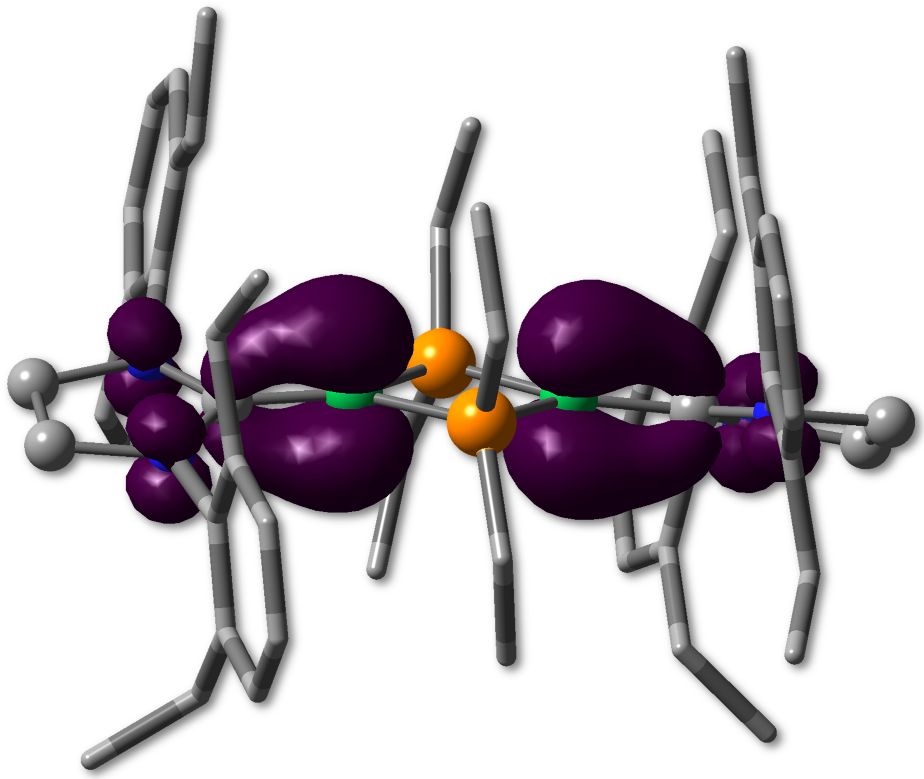

Figure S10. Spin density surface plot of **[3]**$^{+\bullet}$ at the UB3LYP-D3(BJ)/def2SVP level of theory. The Mulliken spin density values are: +0.32 (B); +0.10 (C); +0.06 (N).

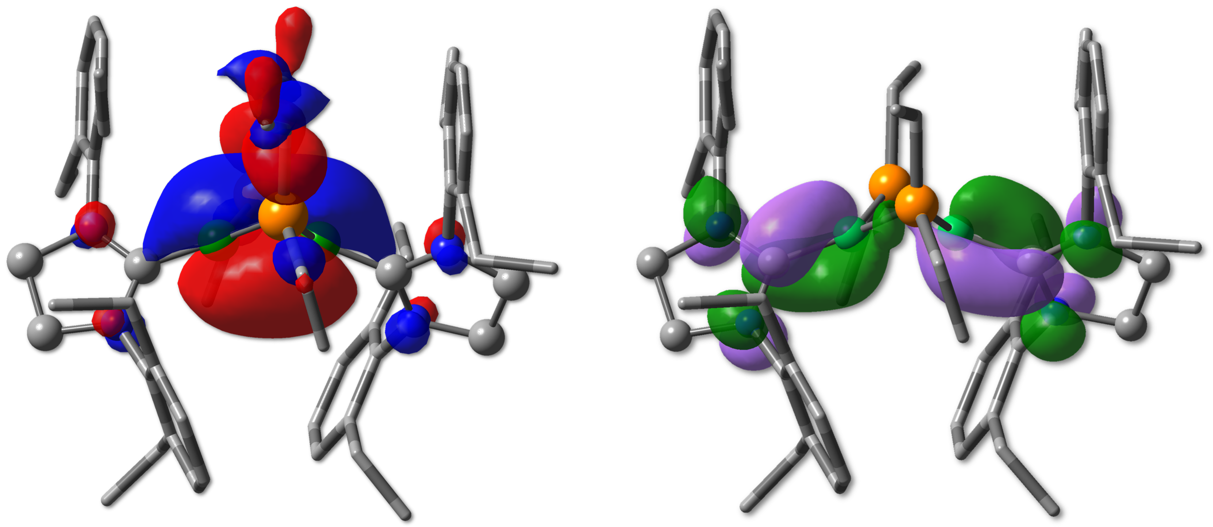

Figure S11. Frontier MOs of **[4]**$^{2+}$ at the B3LYP-D3(BJ)/def2-SVP level of theory. The HOMO-LUMO gap of **[4]**$^{2+}$ is 3.96 eV.

Table S2. NBO bond bending analysis of the B–B bond in **[4]**$^{2+}$.

**NBO**	**List of Centers**		**Hybrid 1**			**Hybrid 2**		
	**Theta**	**Phi**	**Theta**	**Phi**	**Dev**	**Theta**	**Phi**	**Dev**
75. BD(1) B1–B2	101.4	181.5	97.7	137.4	43.6	77.5	46.0	43.5

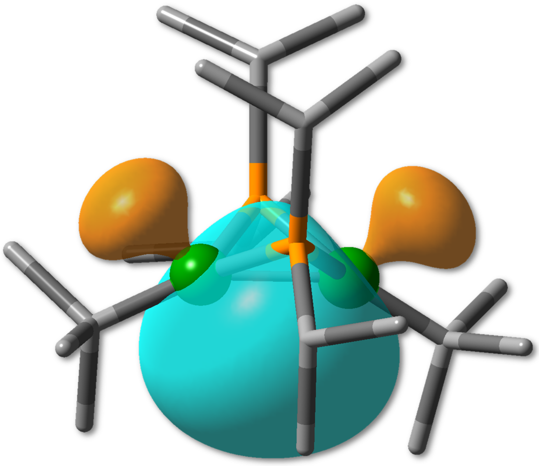

Figure S12. NBO of **D** depicting its bent B–B σ bond.

Table S3. CASSCF and NEVPT2 results for ^{planar}**[4]**$^{2+}$ and **C**.

	^{planar}**[4]**$^{2+}$	**C**
**CASSCF(2,2)/def2-TZVP**		
Electronic energy, E_h	–3040.478917	–1515.195807
Weight of the 2 0 configuration	0.79757	0.85208
Weight of the 0 2 configuration	0.20243	0.14792
Occupation of the HOMO	1.5951	1.7042
Occupation of the LUMO	0.4049	0.2958
HOMO-LUMO orbital overlap (T)	0.5951	0.7042
Biradical character (y_0)	0.12	0.06
**B3LYP-D3(BJ)/def2-SVP**		
HOMO-LUMO gap (eV)	1.74	2.68

Cartesian coordinates

Values in Å

2			
P	-0.156202000	1.331042000	0.237445000
N	3.797949000	-0.661854000	0.134485000
C	2.774303000	0.306415000	0.159171000
B	1.325779000	0.152650000	-0.081372000
C	5.110340000	-0.086365000	0.404093000
H	5.674763000	0.098647000	-0.531468000
H	5.720341000	-0.749337000	1.031588000
N	3.431100000	1.498962000	0.530275000
C	5.485018000	-2.854808000	2.868958000
H	5.615494000	-2.513495000	3.908276000
H	6.262532000	-2.374158000	2.254592000
H	5.678310000	-3.938793000	2.844325000
C	4.074561000	-2.531197000	2.357383000
H	3.341742000	-3.036195000	3.006751000
H	3.871103000	-1.455495000	2.449021000
C	4.743685000	1.216370000	1.101373000
H	4.696278000	1.072049000	2.198585000
H	5.446242000	2.034716000	0.891212000
C	2.861483000	3.770783000	1.195698000
C	3.074640000	2.818638000	0.170596000
C	2.541971000	5.086073000	0.834359000
H	2.352450000	5.830011000	1.609296000
C	2.455312000	5.457579000	-0.506904000
H	2.195461000	6.485019000	-0.772843000
C	3.043687000	3.199809000	-1.194222000
C	2.716289000	4.522860000	-1.509990000
H	2.672580000	4.834420000	-2.553935000
C	2.995652000	3.348716000	2.645324000
H	2.602044000	2.325006000	2.738115000
H	4.069867000	3.262800000	2.890999000
C	2.334004000	4.258517000	3.678195000
H	2.427885000	3.824037000	4.685302000
H	1.260945000	4.393200000	3.469548000
H	2.796486000	5.257421000	3.708486000

C	3.676951000	-2.049197000	-0.129628000
C	3.831684000	-2.972824000	0.931059000
C	3.756132000	-4.343982000	0.650607000
H	3.857715000	-5.060242000	1.470493000
C	3.525338000	-4.802221000	-0.644531000
H	3.453348000	-5.874047000	-0.843917000
C	3.381109000	-3.884066000	-1.684645000
H	3.205223000	-4.237333000	-2.704389000
C	3.463598000	-2.505593000	-1.451044000
C	3.376503000	-1.544334000	-2.612126000
H	2.814316000	-0.652073000	-2.305678000
H	2.795972000	-2.018172000	-3.418952000
C	4.746538000	-1.137649000	-3.167280000
H	4.633952000	-0.446424000	-4.017402000
H	5.312245000	-2.016948000	-3.514450000
H	5.355938000	-0.632370000	-2.404685000
C	-0.323559000	2.845422000	-0.814892000
H	-1.279717000	3.312823000	-0.548779000
H	0.466324000	3.542843000	-0.506390000
C	-0.251238000	2.565630000	-2.308301000
H	-0.420334000	3.486152000	-2.889785000
H	-1.004423000	1.825306000	-2.607432000
H	0.729269000	2.159196000	-2.588368000
C	-0.230001000	2.141171000	1.906780000
H	0.556726000	2.906687000	1.906749000
H	-1.186458000	2.681339000	1.954413000
C	-0.072937000	1.182861000	3.078861000
H	-0.061718000	1.725032000	4.038504000
H	0.862275000	0.608493000	2.993104000
H	-0.897245000	0.454685000	3.107633000
C	3.394977000	2.186561000	-2.256812000
H	4.348897000	1.711112000	-1.973411000
P	0.129269000	-1.313514000	-0.403056000
N	-3.828528000	0.621141000	-0.033608000
C	-2.805289000	-0.338712000	-0.099513000
B	-1.345301000	-0.138284000	-0.064717000
C	-5.147248000	0.012502000	0.092837000

H	-5.468989000	-0.069147000	1.150080000
H	-5.904427000	0.600672000	-0.444689000
N	-3.483657000	-1.568870000	-0.215516000
C	-5.104411000	1.386098000	-3.301543000
H	-5.090411000	0.733429000	-4.189081000
H	-5.758265000	0.916623000	-2.551767000
H	-5.569300000	2.342309000	-3.589767000
C	-3.684981000	1.602730000	-2.760527000
H	-3.067037000	2.038875000	-3.560842000
H	-3.226165000	0.638014000	-2.501898000
C	-4.901521000	-1.365066000	-0.514231000
H	-5.096038000	-1.358830000	-1.604122000
H	-5.511774000	-2.156836000	-0.058056000
C	-2.945700000	-3.908440000	-0.579768000
C	-3.051373000	-2.811759000	0.305673000
C	-2.561623000	-5.152769000	-0.063045000
H	-2.454328000	-6.008951000	-0.730475000
C	-2.306200000	-5.309394000	1.299081000
H	-1.998593000	-6.283352000	1.687330000
C	-2.840140000	-2.969676000	1.697286000
C	-2.453120000	-4.228423000	2.170232000
H	-2.270298000	-4.373548000	3.235293000
C	-3.262831000	-3.707087000	-2.047684000
H	-2.920213000	-2.699676000	-2.331270000
H	-4.361300000	-3.675316000	-2.171886000
C	-2.690949000	-4.748138000	-3.007508000
H	-2.913558000	-4.471949000	-4.049704000
H	-1.596778000	-4.829156000	-2.907592000
H	-3.116514000	-5.749306000	-2.836649000
C	-3.690518000	2.015209000	-0.243243000
C	-3.632638000	2.520930000	-1.561175000
C	-3.569261000	3.907779000	-1.744733000
H	-3.517654000	4.307854000	-2.760929000
C	-3.556129000	4.777118000	-0.652925000
H	-3.492842000	5.856053000	-0.813545000
C	-3.614082000	4.266172000	0.643251000
H	-3.599588000	4.946129000	1.499625000

C	-3.686448000	2.884170000	0.868317000
C	-3.810532000	2.348332000	2.276190000
H	-3.470223000	1.303425000	2.296095000
H	-3.136682000	2.915287000	2.938942000
C	-5.239354000	2.447094000	2.828410000
H	-5.297730000	2.037330000	3.849519000
H	-5.578946000	3.494566000	2.862453000
H	-5.951596000	1.892204000	2.199251000
C	0.391317000	-2.806110000	0.662802000
H	1.285583000	-3.315616000	0.279547000
H	-0.454596000	-3.485509000	0.501793000
C	0.551704000	-2.464601000	2.136839000
H	0.733113000	-3.372703000	2.733868000
H	1.388810000	-1.769567000	2.289417000
H	-0.348886000	-1.975593000	2.529360000
C	0.048008000	-2.137173000	-2.067633000
H	-0.740230000	-2.897713000	-1.972989000
H	0.986188000	-2.685399000	-2.224593000
C	-0.252662000	-1.185229000	-3.217623000
H	-0.306163000	-1.724311000	-4.177648000
H	-1.211281000	-0.670009000	-3.053710000
H	0.521311000	-0.407731000	-3.304538000
C	-3.067930000	-1.797210000	2.624697000
H	-2.400283000	-0.980218000	2.302444000
H	-4.084703000	-1.409191000	2.441330000
C	-2.886732000	-2.059569000	4.115641000
H	-3.088886000	-1.141428000	4.688603000
H	-3.567998000	-2.841847000	4.487015000
H	-1.857338000	-2.372289000	4.351253000
H	2.657166000	1.369789000	-2.197212000
C	3.483640000	2.702547000	-3.688352000
H	3.772607000	1.886396000	-4.368170000
H	4.230464000	3.505717000	-3.794247000
H	2.516889000	3.095264000	-4.040816000

[3]$^{+\bullet}$			
P	-0.222554916	1.346778245	0.219130621
N	3.780695886	-0.509709167	0.270635118
C	2.772278511	0.424780142	0.225096543
B	1.291616343	0.204538062	-0.043937923
C	5.098721316	0.107978209	0.460812697
H	5.633809962	0.171107377	-0.502637709
H	5.712226939	-0.484346431	1.149962165
N	3.352610044	1.640440502	0.529123836
C	5.392339345	-2.508472841	3.139437789
H	5.461550014	-2.130162941	4.170978749
H	6.165017400	-2.002176227	2.540010662
H	5.647290329	-3.579101876	3.155987019
C	3.984879160	-2.282726642	2.568517139
H	3.259378591	-2.807596282	3.209431424
H	3.721201244	-1.216592931	2.617868585
C	4.730576133	1.476196357	1.011958851
H	4.763553803	1.491743103	2.115270269
H	5.370139354	2.285205520	0.635007328
C	2.609386080	3.873801209	1.179372805
C	2.895432980	2.939150498	0.161636601
C	2.223918402	5.165846344	0.796901815
H	1.976812439	5.905626724	1.558653407
C	2.163779725	5.523333945	-0.549811484
H	1.863238280	6.535466610	-0.829576436
C	2.887053103	3.298674526	-1.205972408
C	2.506215472	4.602709315	-1.541241030
H	2.481685741	4.909189505	-2.586801594
C	2.765835376	3.481895634	2.635953191
H	2.440220004	2.436401207	2.752996933
H	3.844709097	3.474026742	2.874941157
C	2.054126039	4.369340402	3.655043388
H	2.176617269	3.958399088	4.668191184
H	0.974418699	4.441598677	3.449913966
H	2.460940612	5.391721531	3.667004990
C	3.714451461	-1.916155163	0.044081748
C	3.827400768	-2.786626655	1.150745480

C	3.823656373	-4.167655622	0.913562162
H	3.894411954	-4.853126837	1.761741247
C	3.719791251	-4.674279331	-0.380683794
H	3.707432444	-5.753746821	-0.546584143
C	3.640868809	-3.800197591	-1.464596019
H	3.579279848	-4.196735034	-2.481369944
C	3.651994814	-2.411594029	-1.276837131
C	3.667414467	-1.499589108	-2.482154952
H	3.209391614	-0.535596053	-2.230724981
H	3.039700700	-1.942734297	-3.270226561
C	5.074432954	-1.275870023	-3.052905699
H	5.036988747	-0.612144913	-3.930823154
H	5.531669492	-2.227443728	-3.364827413
H	5.747008384	-0.817785308	-2.312694431
C	-0.438027992	2.843534122	-0.841112156
H	-1.407087073	3.280819991	-0.572255119
H	0.325544575	3.564538754	-0.524563000
C	-0.353667440	2.577234191	-2.335997942
H	-0.514771077	3.506694505	-2.903134615
H	-1.109482688	1.848959739	-2.657550635
H	0.627836113	2.174535644	-2.617825124
C	-0.384148609	2.121134362	1.891801458
H	0.373590877	2.912806287	1.940253207
H	-1.357722101	2.627789923	1.904125012
C	-0.252154669	1.142321095	3.050100197
H	-0.337586888	1.661886707	4.017271168
H	0.717670175	0.621509096	3.024480626
H	-1.033914680	0.367791647	3.006592413
C	3.311726106	2.291264631	-2.252375707
H	4.284802992	1.867571167	-1.949869914
P	0.195319700	-1.315925692	-0.437148523
N	-3.752322190	0.565134822	0.332755101
C	-2.804451583	-0.421941006	0.167327824
B	-1.315189713	-0.198341862	-0.057992276
C	-5.030480079	0.013330097	0.805027813
H	-5.124361298	0.128779071	1.898024122
H	-5.872964264	0.532531714	0.330019675

N	-3.464137118	-1.619030171	0.293227018
C	-5.134447036	0.615610166	-2.825261272
H	-5.110632863	-0.156664132	-3.609965525
H	-5.596810365	0.170363796	-1.932656958
H	-5.794171095	1.427611250	-3.167197031
C	-3.722416283	1.138544473	-2.533457268
H	-3.299532513	1.548908375	-3.463199278
H	-3.067883997	0.306162312	-2.241737239
C	-4.915944669	-1.451866978	0.410175990
H	-5.408988902	-1.674570488	-0.552972016
H	-5.329390907	-2.133367326	1.165263019
C	-2.771744370	-3.519309879	-1.073433695
C	-2.937360344	-2.939778536	0.200810105
C	-2.318703535	-4.843403724	-1.135201174
H	-2.167539884	-5.323084331	-2.102408605
C	-2.076945464	-5.566646680	0.034204223
H	-1.728548430	-6.599820173	-0.031723413
C	-2.728976392	-3.661980463	1.393300159
C	-2.288749356	-4.987558978	1.285953442
H	-2.105921792	-5.575321482	2.185466033
C	-3.116630816	-2.712766878	-2.309741162
H	-2.550949142	-1.769194767	-2.280413523
H	-4.173662593	-2.405716446	-2.237572752
C	-2.892180953	-3.399413480	-3.652460527
H	-3.172354614	-2.724468172	-4.474822827
H	-1.837260778	-3.678741189	-3.800556051
H	-3.497540315	-4.313438943	-3.751956386
C	-3.687837344	1.924535585	-0.093393650
C	-3.686120021	2.219207152	-1.477322631
C	-3.707663583	3.562220238	-1.870006721
H	-3.698333484	3.801256634	-2.936149116
C	-3.738496545	4.590305411	-0.927089531
H	-3.746944701	5.632508821	-1.253392812
C	-3.764216470	4.282779312	0.431412885
H	-3.796966017	5.086385750	1.171571124
C	-3.750438599	2.951575707	0.872266625
C	-3.857251895	2.664499832	2.354120146

H	-3.510626604	1.641881193	2.563329218
H	-3.174434355	3.338159743	2.896892879
C	-5.276644239	2.871613791	2.902887373
H	-5.317385780	2.649788790	3.980519189
H	-5.605073256	3.912365218	2.759397554
H	-6.008800519	2.226867655	2.394046024
C	0.471240963	-2.822489505	0.590786985
H	1.411240564	-3.271139016	0.246762401
H	-0.323967814	-3.537210151	0.349417905
C	0.518177949	-2.523122177	2.081059468
H	0.725048255	-3.437857207	2.657536087
H	1.299422162	-1.786574303	2.313237092
H	-0.432620260	-2.102912620	2.433747849
C	0.281382788	-2.054071666	-2.136486990
H	-0.486705063	-2.838630882	-2.169061327
H	1.244564389	-2.576569026	-2.202699436
C	0.108498170	-1.050801678	-3.268516154
H	0.160995851	-1.549641111	-4.248749119
H	-0.860588990	-0.533694661	-3.203510280
H	0.888874456	-0.276074124	-3.238694545
C	-3.002424348	-3.001008503	2.729652913
H	-2.565055680	-1.989569558	2.712367169
H	-4.091259096	-2.834975204	2.819841110
C	-2.520960652	-3.756293163	3.964830132
H	-2.731535160	-3.173629799	4.873941633
H	-3.026266027	-4.727987859	4.074385904
H	-1.436860728	-3.941705737	3.928735830
H	2.612385102	1.440238572	-2.210775216
C	3.405835306	2.798057831	-3.687132755
H	3.749720157	1.990946446	-4.351442754
H	4.116504582	3.633546123	-3.781975918
H	2.430093838	3.141410567	-4.064231008

**[4]**$^{2+}$			
B	1.005419506	-0.220533424	0.139345728
B	-0.984317267	-0.272796640	-0.263282917
C	4.791911507	0.526554133	0.065890794
H	5.346297485	0.851300432	-0.823209407
H	5.426328400	-0.183068036	0.619600009
N	-2.958382471	1.347605280	-0.940383707
C	3.608273867	-1.407801351	-1.008933201
N	-3.504005951	-0.436076268	0.220076594
C	3.870391223	-1.433500066	-2.391775981
C	3.950727002	-2.687111121	-3.020374734
H	4.138198351	-2.730195034	-4.095979696
C	3.825270449	-3.864931378	-2.291026247
H	3.896260683	-4.830554120	-2.795976508
C	3.644875390	-3.816670059	-0.905193901
H	3.600441763	-4.748597370	-0.342419393
C	3.546194369	-2.592723998	-0.235578468
C	2.099277114	2.174394915	2.018419762
C	0.846917865	3.749749277	3.918024774
H	0.352984510	4.370918821	4.668277540
C	0.945635712	4.196980646	2.603687000
H	0.538611141	5.173388136	2.330570166
C	1.589092143	3.422741111	1.624695056
C	1.389848336	2.516007881	4.290313394
H	1.316551561	2.192855507	5.328592908
C	2.033910542	1.706073515	3.350793590
N	2.861097901	1.398493600	1.084216057
P	-0.236561234	-1.060085437	1.278436215
C	2.466464296	0.373345372	0.322027727
C	2.678270559	0.383504982	3.721208118
H	3.763224882	0.455728913	3.526657267
H	2.324073493	-0.391064032	3.022404954
C	2.468524092	-0.094608450	5.154716043
H	2.960732166	-1.066304778	5.307273944
H	1.400916369	-0.221006916	5.395537745
H	2.893796600	0.607194343	5.887229560
C	1.772829590	3.966900956	0.226990745

H	0.795003868	4.278802148	-0.169486510
H	2.117131126	3.169233919	-0.443080450
C	2.739750853	5.157387277	0.166724284
H	3.740548171	4.886548963	0.538300572
H	2.382990538	5.996509054	0.782614326
H	2.846888159	5.520516817	-0.866621447
C	-2.483920335	0.240328313	-0.356475437
C	-4.385499639	1.561630246	-0.617183008
H	-4.938243114	1.881335432	-1.508237389
H	-4.467522377	2.355306498	0.141198416
C	-4.806364608	0.197412433	-0.078590842
H	-5.425893628	0.262573692	0.823436524
H	-5.344236445	-0.413713027	-0.821977440
C	-3.485700355	-1.791265618	0.702835294
C	-3.327708191	-2.837288613	-0.235649579
C	-3.329391911	-4.152217003	0.244999901
H	-3.207327166	-4.984190849	-0.447822569
N	3.533735695	-0.144600015	-0.325181178
P	0.311875730	-0.874462668	-1.487529360
C	4.299249684	1.694036521	0.924696454
H	4.785019418	1.755599664	1.908163370
H	4.415347347	2.669076930	0.428553719
C	-3.515774509	-4.416508007	1.603008234
H	-3.513173587	-5.448319260	1.960583435
C	-3.747577671	-3.373753633	2.497647013
H	-3.942118977	-3.602622894	3.545326952
C	-3.765114695	-2.039659767	2.064032777
C	-4.168713802	-0.921942033	3.005833811
H	-3.648516733	0.001266481	2.718292356
H	-5.240646792	-0.714386429	2.832186826
C	-3.976513259	-1.188422006	4.498771108
H	-4.633806786	-1.991160858	4.862915119
H	-4.218737245	-0.286055733	5.078741848
H	-2.940057714	-1.474419666	4.739319533
C	-3.233490129	-2.534851221	-1.718400996
H	-2.302489137	-1.983112304	-1.914102871
H	-4.038972960	-1.828216139	-1.979223418

C	-2.274169031	2.285893329	-1.782497939
C	-3.312636916	-3.736299952	-2.654917740
H	-3.291889759	-3.403015362	-3.702585535
H	-4.242425975	-4.305422505	-2.506525072
H	-2.468129484	-4.427486387	-2.513223916
C	-1.864683801	3.520942497	-1.244523351
C	-1.297326647	4.458634300	-2.120023265
H	-0.970436064	5.424391664	-1.727189973
C	-2.662981761	0.690160160	-3.757622636
H	-2.486072975	-0.143825201	-3.064181694
H	-2.063924936	0.475716978	-4.656487408
C	-2.162999554	1.987673765	-3.158723199
C	-1.600144787	2.961380514	-3.992223526
H	-1.502903901	2.754286781	-5.060662613
C	-1.168265242	4.185612056	-3.480175512
H	-0.733866591	4.932586415	-4.147920385
C	-2.072699134	3.882189701	0.208221933
H	-1.123917987	4.252607261	0.623882164
H	-2.311736604	2.982852744	0.789323806
C	-4.143117720	0.708814065	-4.163027300
H	-4.351052629	1.532164614	-4.862397493
H	-4.807822301	0.835317453	-3.296185689
H	-4.420434915	-0.234306019	-4.657922272
C	-3.156049171	4.949998850	0.414539711
H	-2.898457704	5.886011553	-0.103329191
H	-3.278621651	5.179935503	1.483799555
H	-4.131144164	4.621947914	0.021882220
C	0.006648233	-2.850616006	-3.533693970
H	0.775338895	-2.426120579	-4.194476034
H	-0.961519369	-2.406836228	-3.808140732
H	-0.050208571	-3.927424412	-3.751637503
C	0.354529914	-2.636423402	-2.060054121
H	1.362479230	-3.014423114	-1.844796664
H	-0.338505452	-3.181095935	-1.401492511
C	0.736749713	0.168079103	-2.944089743
H	1.545566726	-0.364865407	-3.461541530
H	-0.123408030	0.156692774	-3.625363292

C	1.151293438	1.593385294	-2.602183708
H	2.084587418	1.607949294	-2.025820613
H	0.386405763	2.114928473	-2.016387552
H	1.319058517	2.168080476	-3.523485707
C	-0.146267986	-2.872844647	1.650217068
H	-1.131953967	-3.294728504	1.412848527
H	0.565242975	-3.293224720	0.923917394
C	0.252532913	-3.219096894	3.085701888
H	0.393941083	-4.305631604	3.183195355
H	-0.528432207	-2.927813600	3.801975225
H	1.191794352	-2.735593071	3.392364173
C	-0.744741946	-0.236746469	2.844171162
H	-1.519155919	-0.885223747	3.274389307
H	0.104552320	-0.275460063	3.537992450
C	-1.252504439	1.189480731	2.681452761
H	-0.520457385	1.832692031	2.181825119
H	-1.467390435	1.626358567	3.666394521
H	-2.179108434	1.219594986	2.094626265
C	4.177391986	-0.191315790	-3.198743504
H	3.838326262	0.705900331	-2.664703105
H	3.609958259	-0.221364749	-4.143012626
C	5.671881778	-0.068163438	-3.534611235
H	6.016133698	-0.923393715	-4.134754698
H	6.292766344	-0.040904662	-2.625568612
H	5.866999696	0.848669987	-4.110857276
C	3.440582862	-2.517024441	1.274663615
H	2.486048982	-2.040180245	1.542526344
H	4.213272685	-1.820075551	1.641044071
C	3.572824018	-3.838225015	2.025014782
H	2.763930116	-4.539926022	1.770717083
H	3.529557417	-3.665515033	3.110007851
H	4.530115139	-4.334773284	1.807253126

^{planar}**[4]**$^{2+}$			
P	0.173866000	-1.382229000	0.221646000
N	-3.735410000	0.630990000	0.412208000
C	-2.827696000	-0.352731000	0.218067000
B	-1.298970000	-0.178936000	-0.078009000
C	-5.033645000	0.093413000	0.870168000
H	-5.855499000	0.572383000	0.323131000
H	-5.165829000	0.305841000	1.940453000
N	-3.440533000	-1.548209000	0.381771000
C	-5.113353000	2.728313000	3.326346000
H	-5.138820000	2.381338000	4.370205000
H	-5.914727000	2.214465000	2.773924000
H	-5.356767000	3.801215000	3.323382000
C	-3.732139000	2.477147000	2.702438000
H	-2.979822000	3.027430000	3.288282000
H	-3.459014000	1.414270000	2.786838000
C	-4.893072000	-1.398584000	0.594681000
H	-5.228674000	-2.024538000	1.430545000
H	-5.427126000	-1.727266000	-0.311909000
C	-2.750927000	-3.788083000	1.069160000
C	-2.965032000	-2.855313000	0.033073000
C	-2.384356000	-5.090073000	0.700999000
H	-2.193388000	-5.837546000	1.470899000
C	-2.289638000	-5.452364000	-0.643045000
H	-2.018648000	-6.476308000	-0.908935000
C	-2.905592000	-3.206423000	-1.333720000
C	-2.559416000	-4.524800000	-1.650755000
H	-2.504064000	-4.836452000	-2.693323000
C	-2.971243000	-3.398849000	2.519222000
H	-2.645084000	-2.356669000	2.663911000
H	-4.060544000	-3.384629000	2.706056000
C	-2.320402000	-4.300965000	3.566533000
H	-2.486301000	-3.892909000	4.574099000
H	-1.233170000	-4.391300000	3.413912000
H	-2.743758000	-5.315906000	3.555027000
C	-3.615585000	2.037961000	0.170094000
C	-3.641477000	2.930307000	1.261629000

C	-3.611405000	4.305343000	0.985948000
H	-3.621253000	5.012883000	1.818403000
C	-3.564993000	4.777259000	-0.323657000
H	-3.538945000	5.851575000	-0.517357000
C	-3.565840000	3.875809000	-1.389757000
H	-3.553794000	4.248329000	-2.416852000
C	-3.606956000	2.493676000	-1.168425000
C	-3.712824000	1.554096000	-2.349679000
H	-3.082144000	0.668413000	-2.196520000
H	-3.306712000	2.064487000	-3.236148000
C	-5.148384000	1.110134000	-2.658998000
H	-5.172341000	0.476749000	-3.558675000
H	-5.800720000	1.977742000	-2.837654000
H	-5.587067000	0.531032000	-1.833407000
C	0.391595000	-2.894497000	-0.800910000
H	1.327861000	-3.350910000	-0.456249000
H	-0.413431000	-3.579961000	-0.509639000
C	0.411162000	-2.667497000	-2.304524000
H	0.591746000	-3.618839000	-2.826007000
H	1.203781000	-1.968275000	-2.599866000
H	-0.541327000	-2.263040000	-2.667986000
C	0.243854000	-2.077549000	1.926254000
H	-0.539106000	-2.845092000	1.967525000
H	1.199429000	-2.611605000	1.997425000
C	0.088437000	-1.051695000	3.040749000
H	0.129454000	-1.542056000	4.024820000
H	-0.872179000	-0.517789000	2.970263000
H	0.886431000	-0.293766000	3.007484000
C	-3.221552000	-2.177643000	-2.400230000
H	-4.182599000	-1.696173000	-2.156074000
P	-0.174198000	1.312576000	-0.548075000
N	3.713584000	-0.620093000	0.432052000
C	2.820638000	0.371273000	0.194524000
B	1.296197000	0.151396000	-0.086342000
C	5.009200000	-0.078552000	0.894265000
H	5.093336000	-0.201885000	1.984681000
H	5.838105000	-0.617857000	0.420185000

N	3.458891000	1.559044000	0.300493000
C	5.252635000	-0.976705000	-2.634411000
H	5.291642000	-0.306767000	-3.506794000
H	5.675192000	-0.432718000	-1.777416000
H	5.911171000	-1.834324000	-2.837285000
C	3.813470000	-1.439315000	-2.371417000
H	3.430439000	-1.923068000	-3.282294000
H	3.169945000	-0.565069000	-2.200953000
C	4.912460000	1.385329000	0.490747000
H	5.431797000	1.613855000	-0.454834000
H	5.288621000	2.070324000	1.259058000
C	2.818165000	3.283468000	-1.306354000
C	2.970872000	2.879223000	0.037131000
C	2.408158000	4.601454000	-1.543417000
H	2.273714000	4.954140000	-2.565846000
C	2.200126000	5.484290000	-0.481862000
H	1.888943000	6.510969000	-0.686065000
C	2.815802000	3.765112000	1.123683000
C	2.412856000	5.075987000	0.834880000
H	2.269800000	5.790054000	1.645516000
C	3.138209000	2.318811000	-2.432229000
H	2.495453000	1.429637000	-2.335190000
H	4.162380000	1.938459000	-2.281367000
C	3.020716000	2.867340000	-3.850256000
H	3.291244000	2.090348000	-4.580027000
H	1.996330000	3.196260000	-4.084609000
H	3.692219000	3.723567000	-4.012895000
C	3.610343000	-2.016243000	0.125396000
C	3.684608000	-2.421050000	-1.227928000
C	3.692892000	-3.793929000	-1.501556000
H	3.748586000	-4.126528000	-2.540593000
C	3.632346000	-4.735331000	-0.473144000
H	3.634848000	-5.801698000	-0.708222000
C	3.582522000	-4.314135000	0.854451000
H	3.552291000	-5.053120000	1.658803000
C	3.589695000	-2.950438000	1.182015000
C	3.644051000	-2.542230000	2.638745000

H	3.336543000	-1.491398000	2.751517000
H	2.908527000	-3.137477000	3.203738000
C	5.027442000	-2.766950000	3.268530000
H	5.032666000	-2.449906000	4.322197000
H	5.305458000	-3.830893000	3.235358000
H	5.814869000	-2.210141000	2.738827000
C	-0.373913000	2.848429000	0.436058000
H	-1.321230000	3.301322000	0.121594000
H	0.421631000	3.535596000	0.125413000
C	-0.344721000	2.596403000	1.935467000
H	-0.516926000	3.533010000	2.484830000
H	-1.119443000	1.879558000	2.238671000
H	0.622396000	2.187685000	2.257053000
C	-0.284832000	1.932400000	-2.284214000
H	0.498988000	2.695733000	-2.381955000
H	-1.239507000	2.472522000	-2.344959000
C	-0.174161000	0.863777000	-3.363395000
H	-0.237682000	1.319319000	-4.362689000
H	0.780132000	0.319995000	-3.303888000
H	-0.981295000	0.120994000	-3.283185000
C	3.113097000	3.313502000	2.540510000
H	2.749641000	2.280611000	2.668119000
H	4.210013000	3.244776000	2.656681000
C	2.571655000	4.200085000	3.659577000
H	2.792918000	3.751696000	4.638970000
H	3.032297000	5.198848000	3.649886000
H	1.481639000	4.329970000	3.586188000
H	-2.476780000	-1.366518000	-2.327519000
C	-3.270963000	-2.682787000	-3.838178000
H	-3.536627000	-1.860928000	-4.519254000
H	-4.023738000	-3.475405000	-3.964372000
H	-2.300610000	-3.083965000	-4.168219000

C

B	-0.082000920	1.278840273	-0.106941378
P	-1.465117481	-0.000023574	0.000078256
P	1.315064705	0.000036395	0.000061533
B	-0.081943777	-1.278833923	0.107061641
C	2.451816807	0.285659737	1.497155078
C	2.451824523	-0.285536696	-1.497028007
C	3.563935174	1.309093654	1.251574828
C	2.980365859	-0.976667666	2.179393089
C	2.980517606	0.976820741	-2.179098909
C	3.563824116	-1.309117848	-1.251521509
C	-0.152815207	-2.878757001	0.104271548
C	0.989043523	-3.550915493	0.884608806
C	-0.068352987	-3.338714506	-1.370959883
C	-1.484597977	-3.377223009	0.698492976
C	-2.658217091	-0.024815082	1.472610234
C	-1.853030556	-0.092782925	2.770190568
C	-3.668973799	1.123793176	1.497166339
C	-2.658230913	0.024735268	-1.472444406
C	-1.853048252	0.092611218	-2.770031259
C	-3.669025182	-1.123840040	-1.496946503
C	-0.152950862	2.878759726	-0.104506206
C	-0.067492116	3.339108584	1.370545447
C	0.988323419	3.550777372	-0.885820198
C	-1.485176183	3.376991436	-0.697932805
H	-0.883229939	2.915684286	1.976386811
H	-0.135427636	4.440556171	1.440963873
H	0.882670064	3.033400765	1.836262644
H	1.978939107	3.237060800	-0.526558773
H	0.933225272	4.649547682	-0.787014879
H	0.934029410	3.315072210	-1.959867114
H	-1.588173607	3.085555893	-1.755283068
H	-2.352196195	2.973459144	-0.155821830
H	-1.550861182	4.478542160	-0.649852660
H	-1.148135991	-0.937721591	2.766854132
H	-2.523765138	-0.204574477	3.638255182
H	-1.255702824	0.821895444	2.914317146

H	-4.271578202	1.177560087	0.578498812
H	-3.165348604	2.094317273	1.627123550
H	-4.367579850	1.004128652	2.342307238
H	-3.208683315	-0.974330475	1.361466845
H	-1.148124772	0.937525816	-2.766739967
H	-2.523782262	0.204380792	-3.638099170
H	-1.255751926	-0.822096092	-2.914111952
H	-4.271610667	-1.177563543	-0.578263895
H	-3.165433632	-2.094382860	-1.626891755
H	-4.367645738	-1.004174987	-2.342075394
H	-3.208665736	0.974273985	-1.361341954
H	-1.586872064	-3.086079577	1.755993731
H	-1.550253464	-4.478764478	0.650162377
H	-2.352018878	-2.973595513	0.157098237
H	0.881484834	-3.032864820	-1.837244659
H	-0.884513263	-2.915143562	-1.976131954
H	-0.136315098	-4.440144688	-1.441624525
H	0.935528812	-3.315440608	1.958746253
H	1.979389860	-3.237101494	0.524686284
H	0.933895375	-4.649665832	0.785608268
H	1.722635525	-0.743197955	-2.186927975
H	4.038445439	-1.586884092	-2.207800922
H	3.183110650	-2.232663151	-0.792042005
H	4.355652147	-0.909335332	-0.601142598
H	3.521208625	0.705862293	-3.102090936
H	3.679231080	1.537315308	-1.541552144
H	2.163064230	1.653160426	-2.460380345
H	1.722655464	0.743470254	2.186985269
H	4.355730497	0.909162815	0.601246878
H	4.038570203	1.586892263	2.207837927
H	3.183336990	2.232639719	0.792006899
H	3.521067393	-0.705647887	3.102361075
H	3.679035480	-1.537313128	1.541931914
H	2.162839197	-1.652885995	2.460745610

**D**			
B	-0.117725000	1.024144000	-0.410801000

P	-1.462765000	0.110697000	0.537131000
P	1.331217000	-0.072190000	0.120631000
B	-0.243659000	-0.992512000	-0.395573000
C	2.054654000	-0.083718000	1.861442000
C	2.748187000	-0.278957000	-1.102606000
C	3.152340000	0.959705000	2.082015000
C	2.430656000	-1.472598000	2.385287000
C	3.492483000	1.009369000	-1.475488000
C	3.768181000	-1.349096000	-0.688001000
C	-0.418111000	-2.385940000	-1.206584000
C	0.733800000	-3.357059000	-0.884656000
C	-0.417511000	-2.127245000	-2.726851000
C	-1.724759000	-3.100621000	-0.816813000
C	-1.804142000	0.281458000	2.396757000
C	-1.378562000	-0.969117000	3.166551000
C	-1.205539000	1.578079000	2.947110000
C	-3.190763000	0.304935000	-0.184708000
C	-3.206786000	0.201594000	-1.711839000
C	-4.233243000	-0.640783000	0.428537000
C	-0.138912000	2.473436000	-1.137507000
C	0.918427000	3.376335000	-0.465841000
C	0.148463000	2.350516000	-2.646146000
C	-1.490051000	3.196206000	-0.963906000
H	0.694393000	3.505639000	0.605818000
H	0.934637000	4.380921000	-0.926795000
H	1.931562000	2.960434000	-0.537070000
H	1.097098000	1.832684000	-2.848489000
H	0.198487000	3.343192000	-3.130336000
H	-0.640421000	1.771498000	-3.151992000
H	-2.312653000	2.681647000	-1.477826000
H	-1.761364000	3.289570000	0.100118000
H	-1.438774000	4.217638000	-1.381261000
H	-1.893197000	-1.865162000	2.786340000
H	-1.614162000	-0.868333000	4.239478000
H	-0.298696000	-1.155129000	3.077241000
H	-1.604977000	2.457467000	2.419302000
H	-0.111606000	1.609857000	2.836994000

H	-1.439855000	1.688771000	4.019365000
H	-2.897820000	0.370729000	2.475019000
H	-2.369626000	0.732652000	-2.179584000
H	-4.147045000	0.619827000	-2.106254000
H	-3.149411000	-0.845607000	-2.036932000
H	-4.349985000	-0.507007000	1.513089000
H	-3.980185000	-1.694777000	0.245759000
H	-5.218544000	-0.450356000	-0.028438000
H	-3.463311000	1.336519000	0.096050000
H	-1.770959000	-3.285382000	0.268931000
H	-1.807463000	-4.076743000	-1.327356000
H	-2.612072000	-2.516106000	-1.091487000
H	0.521274000	-1.650162000	-3.050797000
H	-1.237186000	-1.458715000	-3.026530000
H	-0.524132000	-3.070785000	-3.293411000
H	0.832924000	-3.526284000	0.199781000
H	1.695895000	-2.980707000	-1.255680000
H	0.565255000	-4.338631000	-1.362857000
H	2.215587000	-0.634052000	-1.999771000
H	4.419387000	-1.586310000	-1.545371000
H	3.302037000	-2.282742000	-0.351949000
H	4.420234000	-0.985504000	0.119811000
H	4.279858000	0.777449000	-2.212449000
H	3.983514000	1.465716000	-0.603844000
H	2.832221000	1.759758000	-1.922544000
H	1.173176000	0.246574000	2.427191000
H	4.086576000	0.689139000	1.567581000
H	3.386969000	1.045260000	3.156584000
H	2.842328000	1.953586000	1.724518000
H	2.562689000	-1.440960000	3.480176000
H	3.370150000	-1.841708000	1.952512000
H	1.644460000	-2.210120000	2.162284000

References

[1] S. Stoll, A. Schweiger, *J. Magn. Reson.* **2006**, *178*, 42-55.

[2] M. Arrowsmith, J. Böhnke, H. Braunschweig, M. A. Celik, T. Dellermann, K. Hammond, *Chem. Eur. J.* **2016**, *22*, 17169-17172.

[3] S. Tanaka, M. Takashina, H. Tokimoto, Y. Fujimoto, K. Tanaka, K. Fukase, *Synlett* **2005**, 2325–2328.

[4] M. Brookhart, B. Grant, A. F. Volpe Jr., *Organometallics* **1992**, *11*, 3920–3922.

[5] G. M. Sheldrick, *Acta Crystallogr. A* **2015**, *71*, 3-8.

[6] G. M. Sheldrick, *Acta Crystallogr. A* **2008**, *64*, 112-122.

[7] a) S. H. Vosko, L. Wilk, M. Nusair, *Can. J. Phys.* **1980**, *58*, 1200–1211; b) C. Lee, W. Yang, R. G. Parr, *Phys. Rev. B* **1988**, *37*, 785–789; c) A. D. Becke, *J. Chem. Phys.* **1993**, *98*, 5648–5652; d) P. J. Stephens, F. J. Devlin, C. F. Chabalowski, M. J. Frisch, *J. Phys. Chem.* **1994**, *98*, 11623–11627.

[8] S. Grimme, J. Antony, S. Ehrlich, H. Krieg, *J. Chem. Phys.* **2010**, *132*, 154104.

[9] S. Grimme, S. Ehrlich, L. Goerigk, *J. Comput. Chem.* **2011**, *32*, 1456–1465.

[10] F. Weigend, R. Ahlrichs, *Phys. Chem. Chem. Phys.* **2005**, *7*, 3297.

[11] a) R. Seeger, J. A. Pople, *J. Chem. Phys.* **1977**, *66*, 3045–3050; b) R. Bauernschmitt, R. Ahlrichs, *J. Chem. Phys.* **1996**, *104*, 9047–9052.

[12] R. S. Mulliken, *J. Chem. Phys.* **1955**, *23*, 1833–1840.

[13] F. L. Hirshfeld, *Theor. Chim. Acta* **1977**, *44*, 129–138.

[14] P. Löwdin, *J. Chem. Phys.* **1950**, *18*, 365–375.

[15] T. Lu, F. Chen, *J. Theor. Comput. Chem.* **2012**, *11*, 163–183.

[16] A. V. Marenich, S. V. Jerome, C. J. Cramer, D. G. Truhlar, *J. Chem. Theory Comput.* **2012**, *8*, 527–541.

[17] a) I. Mayer, *Chem. Phys. Lett.* **1983**, *97*, 270–274; b) I. Mayer, *Int. J. Quantum Chem.* **1984**, *26*, 151–154.

[18] B. O. Roos, in *Adv. Chem. Phys. Ab Initio Methods Quantum Chem. Part 2, Vol. 69* (Ed.: K.P. Lawley), John Wiley & Sons, **1987**, pp. 399–445.

[19] a) C. Angeli, R. Cimiraglia, S. Evangelisti, T. Leininger, J.-P. Malrieu, *J. Chem. Phys.* **2001**, *114*, 10252–10264; b) C. Angeli, R. Cimiraglia, J.-P. Malrieu, *Chem. Phys. Lett.* **2001**, *350*, 297–305; c) C. Angeli, R. Cimiraglia, J.-P. Malrieu, *J. Chem. Phys.* **2002**, *117*, 9138–9153.

[20] F. Neese, *J. Comput. Chem.* **2003**, *24*, 1740–1747.

[21] a) K. Yamaguchi, *Chem. Phys. Lett.* **1975**, *33*, 330–335; b) S. Yamanaka, M. Okumura, M. Nakano, K. Yamaguchi, *J. Mol. Struct.* **1994**, *310*, 205–218; c) M. Nakano, *Top. Curr. Chem.* **2017**, *375*, 47.

[22] F. Weinhold, C. R. Landis, E. D. Glendening, *Int. Rev. Phys. Chem.* **2016**, *35*, 399–440.

[23] E. D. Glendening, C. R. Landis, F. Weinhold, *J. Comput. Chem.* **2019**, jcc.25873.

[24] F. Neese, *Wiley Interdiscip. Rev. Comput. Mol. Sci.* **2012**, *2*, 73–78.

[25] T. Lu, F. Chen, *J. Comput. Chem.* **2012**, *33*, 580–592.

[26] M. J. Frisch, G. W. Trucks, H. B. Schlegel, G. E. Scuseria, M. A. Robb, J. R. Cheeseman, G. Scalmani, V. Barone, B. Mennucci, G. A. Petersson, H. Nakatsuji, M. Caricato, X. Li, H. P. Hratchian, A. F. Izmaylov, J. Bloino, G. Zheng, J. L. Sonnenberg, M. Hada, M. Ehara, K. Toyota, R. Fukuda, J. Hasegawa, M. Ishida, T. Nakajima, Y. Honda, O. Kitao, H. Nakai, T. Vreven, J. A. Montgomery Jr., J. E. Peralta, F. Ogliaro, M. Bearpark, J. J. Heyd, E. Brothers, K. N. Kudin, V. N. Staroverov, R. Kobayashi, J. Normand, K. Raghavachari, A. Rendell, J. C. Burant, S. S. Iyengar, J. Tomasi, M. Cossi, N. Rega, J. M. Millam, M. Klene, J. E. Knox, J. B. Cross, V. Bakken, C. Adamo, J. Jaramillo, R. Gomperts, R. E. Stratmann, O. Yazyev, A. J. Austin, R. Cammi, C. Pomelli, J. W. Ochterski, R. L. Martin, K. Morokuma, V. G. Zakrzewski, G. A. Voth, P. Salvador, J. J. Dannenberg, S. Dapprich, A. D. Daniels, Ö. Farkas, J. B. Foresman, J. V Ortiz, J. Cioslowski, D. J. Fox, *Gaussian 16, Revision B.01*, Gaussian, Inc., Wallingford CT, **2016**.
